# The Theoretical Model of Decision-Making Behaviour Geospatial Analysis Using Data Obtained from the Games of Chess

**DOI:** 10.3390/ijerph191912353

**Published:** 2022-09-28

**Authors:** Agnieszka Szczepańska, Rafał Kaźmierczak

**Affiliations:** 1 Department of Socio-Economic Geography, Institute of Spatial Management and Geography, Faculty of Geoengineering, University of Warmia and Mazury in Olsztyn, Prawocheńskiego 15, 10-724 Olsztyn, Poland; 2 Department of Spatial Analysis and Real Estate Market, Institute of Spatial Management and Geography, Faculty of Geoengineering, University of Warmia and Mazury in Olsztyn, Prawocheńskiego 15, 10-724 Olsztyn, Poland

**Keywords:** personality traits, geographic differentiation of personality, decision-making, chess

## Abstract

The game of chess offers a conducive setting to explore basic cognitive processes, including decision-making. The game exercises analytical cause-and-effect thinking skills regardless of the level of play. Moreover, chess portals provide information on the chess games played and serve as a vast database. The numbers of games played thus have the potential to be analyzed comprehensively, including for purposes other than analyzing chess matches only. The primary objective of this study is to develop a methodology for using information obtained from chess games for geospatial social analysis. The assumption is that the methodology will allow for general geographical variation in personality inference in the future, relying on big data from chess databases. Future large-scale studies of the geographical differentiation of personality traits using the developed methodology may be applicable in a number of ways. The results can be used wherever cross-sectional social analyses are needed in the context of personality traits (decision-making) to better understand their geographical background. In turn, the geographical distribution of these traits is accompanied by a range of important social, educational, health, political and economic implications.

## 1. Introduction

Social analyses are venturing into ever new areas. In recent years, studies concerning the links between the psychological characteristics of people and the characteristics of the places they live in have been advancing. This is known as geographical psychology, which aims to comprehend psychological phenomena based on their spatial distribution [1]. One of the aims of research in geographical psychology is the geographical organization of personality traits and mapping geographical variation in psychological phenomena across regions [2]. This is because, according to observations, there is geographical variation in the way people think, feel and behave [3]. At the same time, there is a growing interest in personality geography linked to geographical psychology. Personality traits are defined as “dimensions of individual differences in tendencies to show consistent patterns of thoughts, feelings, and actions” [4] p. 25.

To better understand the geographical origins of variation in personality traits, further large-scale studies are required. As Allik & McCrae [5] p.25 note, past research into the link between psychological variables and geography focused mainly on emotion and aggression, with the result that “We are still far from a complete geography of personality, in which the distribution of traits might be mapped like rainfall or population density”. This is largely related to the unavailability of cross-sectional data (big data studies). On the other hand, we have a number of social analyses using data from chess games, but they have been used in research in a completely different context.

In contrast, no items were found in the literature referring to cross-sectional studies on personality traits using the information obtained from chess games in terms of geographical location. Since chess is a deterministic game of individuals, and chess games are accurately documented and digitally available (every game streamed or played over the Internet is recorded), chess data are a potential source of information that can be used to study regional differences in traits in strategic behaviour, as well as individual personality traits—decision-making ability under the pressure of time.

In the case of a chess game, the individual decides on a plan of action to achieve the intended outcome. This is directly reflected in the structure of any chess game. This is directly reflected in the structure of any chess game. Decisions in a chess game are made every half move (a full move consists of moves made by both players). A game is an extensive decision-making process test. For example, a game of 50 moves comprises 100 decisions made by the players. These are not random decisions as they are based on knowledge and a cause-and-effect analysis performed by both parties in a chess game. Naturally, the decision quality is directly correlated with the players’ skills, but also with their personality traits, manifesting themselves in their behaviour and in the choices made on the chessboard (e.g., a tendency to take a risk, expressed in the choice of move variants, aggressive play scenarios, etc.). This is particularly noticeable in beginners and intermediate-level players. Consequently, analysing a chess game allows us to evaluate the decision-making process (both short and long term). The primary objective of the manuscript is to present the concept of social geospatial analysis using data obtained from the games of chess.

The authors would like to stress the huge potential and universal nature of the information from the database of all chess games played. Its resources allow for a large number of multi-variant social analyzes, taking into account the spatial locations—data from the chess game played can be used for social analyzes of personality traits for, basically, the whole world. The number of players around the world creating a study sample of an unprecedented size, which—combined with the small scale of the survey studies conducted so far (mainly based on the Big Five)—considerably increases the reliability and, primarily, the speed of inference (for example, large scale data can be obtained in one day, concerning various nations, and, therefore, an analysis of the geographical distribution of personality can be performed). The analyzes do not have a regional range, but they concern the whole world.

## 2. Literature Review

As Rentfrow et al. [6] point out, studies indicate that personality traits are unevenly distributed geographically, and a common theme that emerges from all the research is that there are strong links between the places in which people live and their attitudes, motivations and well-being. Hence, there is a widespread belief that people’s personality traits do vary according to geographical location, and the existence of real differences in personality traits across geographical regions is becoming increasingly evident [7]. It is important to note that national stereotypes surrounding personality traits that tend to be associated with the ‘typical’ member of a given culture are deeply rooted in society. For example, Southerners are perceived as more emotional and expressive [8,9,10], Italians are passionate, Americans are aggressive, and Finns are taciturn [11]. These views towards personality differences are not always true nor scientifically proven. However, there are few studies investigating this issue on a global scale [5,12,13]. Most often, such studies look at variations at the regional scale limited to a selected country [6,14,15].

The interest in the geographical distribution of personality stems from the establishment of a model commonly used to assess personality traits. Previously, according to Campbell [16], the difficulty in incorporating personality into the field of geography was that personality was described in a variety of ways, and there was no uniform standard of description. Currently, the most widely used is The Five Factors Model [4,17], although other models exist as well: Abridged Big Five Dimensional Circumplex [18], the Six-Factor model [19,20] and the Three-Factor model [21]. The most frequently used research is the Five-Factor Model (also known as the Big Five), which also seems to be culturally universal; it has been evidenced in the course of international projects that it is effective in more than 50 cultures in Europe, Asia, Africa and the Americas [5,22,23,24,25]. The studies conducted under The Five Factors Model demonstrated the existence of statistically measurable geographical variation in personality factors, although given the technique of conducting these studies (a questionnaire for diagnosing personality traits), they were not large-scale studies, and there is no global data available.

A decision-making process should take into account a range of complex factors, including individual predispositions and personality traits. For decades, researchers from different disciplines have studied decision-making styles and personality traits. For example, The Big Five model was used to investigate the role of personality types in predicting decision-making styles [26,27,28]. The existence of links between personality and decision-making in many different areas of life is also supported by other studies for example: [29,30,31,32,33,34,35,36], and a detailed literature review in this area was presented by Mendes et al. [37].

It is interesting to use chess in seeking links between personality traits and the decisions taken. The game of chess offers a conducive environment for studying basic cognitive processes, including decision-making [38,39,40]. To cognitive psychologists, chess is what the fruit fly is to geneticists [41]. Chess is an intellectually complex and strategically demanding game in which the player is the agent of decision-making processes—deciding what move to make next and when to make it, responding to changes affecting the chessboard [42]. Decisions made by the players depend on their personality traits [43], which has been confirmed in the few studies on the issue, usually conducted on a small sample. Their findings show that strong chess players do not seem to be social eccentrics and personality factors that seemed to be irrelevant for chess skills in males were important among the best female players [44]. According to the findings of other studies, among all analysed personality dimensions, only domain-specific performance motivation and emotion expression control determine the playing strength [45]. The findings of a study conducted by Blanch and Llaveria [46] show that chess players scored lower in neuroticism and higher in expressive suppression compared with the general population. Other studies demonstrated that men are more impatient and women are more inconsistent and perform worse under time pressure [47]. Children, in turn, who score higher on openness and extraversion are more likely to play chess [48]. The findings indicate that personality plays an important role in decisions made by players during a chess game. Furthermore, inference based on decision-making in chess games can be applied to future decisional behaviour [49]. The game of chess requires problem-solving skills, so it should require planning skills to calculate multiple moves ahead [50]. The results of the study confirmed that chess players displayed better planning performance than non-chess players, which was evident most prominently in difficult tasks. Time pressure is also an important factor influencing the decision-making process [51,52,53,54,55], although chess masters performed equally well in fast and slow decisions and thus coped well under time pressure [56].

Nowadays, chess is used to improve artificial intelligence algorithms and machine learning methods. An example is the 2017 computer program AlphaZero. Based on chess results, in 2020, Miric et al. [57] evaluated the role of experience in developing quality decision-making skills when compared to the performance of a mature AI as a benchmark. Dilmaghani [58] conducted the first worldwide quantitative investigation based on a cross-sectional analysis of Elo Rating Ratio data into the extent of the gender gap in competitive chess. In another study, Dilmaghani [59] analysed differences between genders in terms of the effect of time constraints on chess performance. Similar research was conducted by Stafford [60]. In contrast, Dreber et al. [61] investigated the relationship between attractiveness and risk-taking in chess. Linnemer and Visser [62] developed a theoretical model to obtain predictions on participation decisions and game outcomes, relying on reviewed game results. Grabner et al. [45] studied individual differences in the extent of chess knowledge. Others analysed the effect of COVID−19 confinement on behavioural, psychological and training patterns of chess players based on their gender, level of education and level of chess played [63]. Vishkin analysed the gender-equality paradox in chess participation across different countries [64]. Summing up, analyses concerning the players and chess games are performed on various planes, which shows that chess makes an interesting study object.

## 3. Data Sources

**Figure 1:**
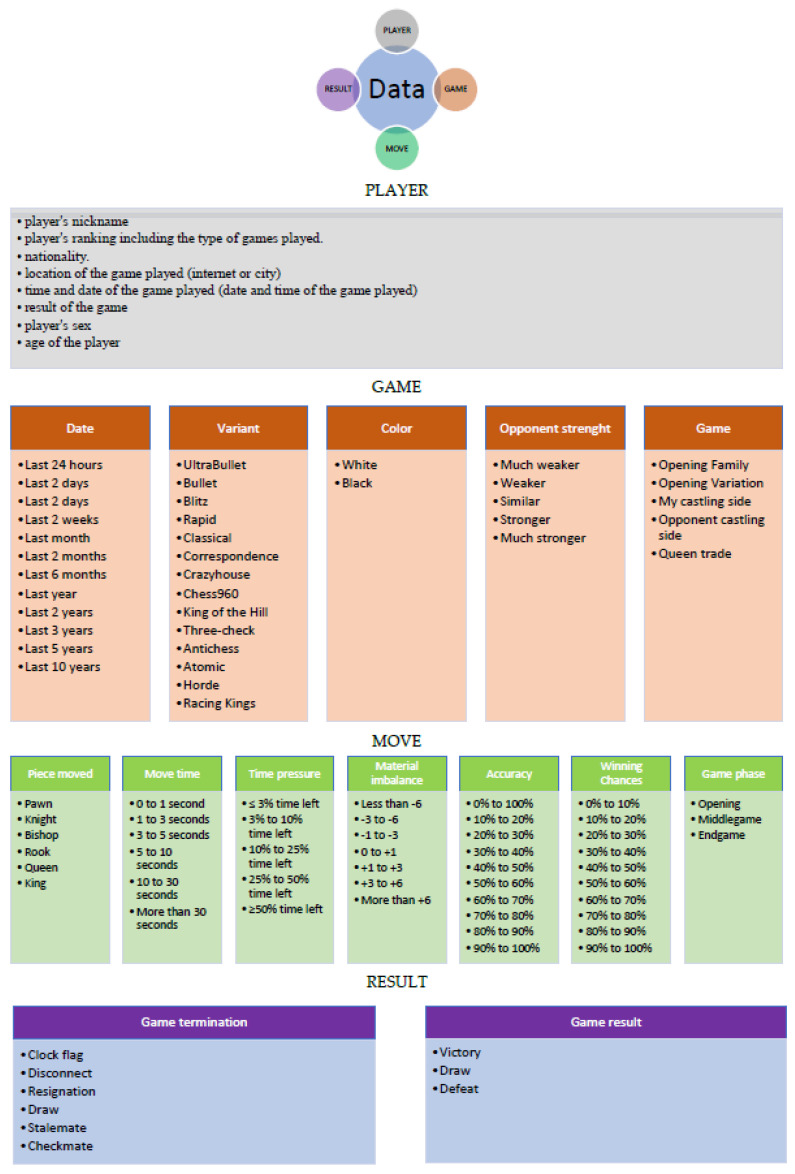
Information retrievable from online chess databases. Source: own study.

The database is the basis of any statistical analysis. The database will form the foundation for future statistical analyses and the conclusions drawn from them. Online chess websites that enable online play and stream live tournament games (in the classic competition format) will serve as a source of information. The most popular websites include:

- https://www.chess.com (accessed on 10 August 2022) (by 2014, the portal had hosted 1 trillion games.) Chess.com was officially launched on 17 May 2007, which means that an average of 4.18 games are started every second, around the clock (a significant increase during the pandemic).
- https://lichess.org/ (accessed on 10 August 2022) (in October 2021 there were 88,092,721 games in the database)
- https://chess24.com/pl (accessed on 10 August 2022) (the tree of openings contains 412,493,642 games)
- https://en.chessbase.com/ (accessed on 10 August 2022).
- https://chesstempo.com/ (accessed on 10 August 2022) (3 million games in the database).
- These can be used to create a database containing four groups of information which, in turn, allow for the filtering of the information obtained in order to better select the research sample. This includes information related to the player (PLAYER), general data about the game played (GAME), the move made (MOVE) and the result (RESULT). Figure 1 shows the possible settings for the filtration of the extracted information.

Since not all of the information is available on chess portals, some of it will be supplemented by extracting data from other online sources. For this purpose, we will employ Web Scraping algorithms. Web Scraping is a technique for extracting data from web pages that replaces manual, repetitive typing or copying and pasting. When scraping pages, links are often extracted as the first step, and specific data can then be extracted from them. In our project, links will be generated automatically, based on the user/player name. Web indexing (of chess websites) will involve the automatic extraction of data from a web page and the extraction of the hyperlinks (links) it contains. The process of data extraction will consist of the creation of code and sending a request to the server hosting the indicated player’s page. The code created will retrieve the source code of the page in the same way as a web browser. Instead of displaying the entire page, it will be filtered to find predefined HTML elements (or other languages: CSS, PHP, javascript). As a result, the database will be supplemented with additional information.

## 4. The Model of Social Geospatial Analysis Using Data Obtained from Games of Chess

**Figure 2:**
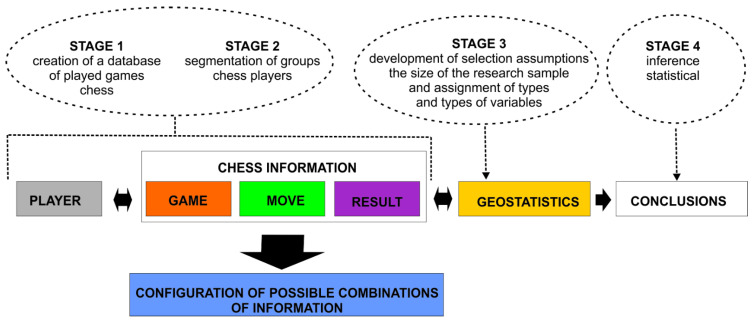
The model of social geospatial analysis using data obtained from the games of chess. Source: own study.

The general methodological assumptions can be split into two main areas. The first area, related to analyses of chess games played, should be juxtaposed with the second area—statistical inference, which is dependent on the selection of the research sample. The following is a proposed template for a model of social-spatial analyses using chess game data based on information obtained from chess games (Figure 2).

Four main stages can be identified in the adopted model. The first two involve creating a standardised database of players and the course of the chess games played by them. The model assumes that this will provide the base for selecting the players meeting a specified criterion. This will give the prevalence, which will create the basis for statistical inference. The criteria for selecting a study sample can be based on the information in the player description (see Figure 1), e.g., sex, age, etc. The chess games played by the players are analysed in the next step. This involves an analysis of the elements concerning the Game Move Result in Figure 2. This information will constitute the attributes of the database and, in consequence, it will help to better characterise a player, understood as a subject of behavioural analyses.

The analysis starts with a single player, for whom a range of correlations between the attributes shown in Figure 6 is generated. Correlations between attributes are determined for individual games played by a player. The process is reiterated for each player assigned to a selected (based on age, sex, etc.) study group. (Stage 3 on Figure 2).

With a representative sample for a defined problem, one can develop pooled statistics for a group of players selected on the basis of the spatial location. This allows for creating geospatial analyses, based on which one can characterise various nations, which—in the case of chess—is particularly highlighted in decision-making processes.

The assumptions made about the individual elements of the model are discussed below: STAGE 1, STAGE 2, STAGE 3, STAGE 4, Chess information (game, move, result), Configuration of possible combinations of information and Geostatistics.

### 4.1. Stage 1—The Creation of a Database of Played Chess Games

Stage 1 will form the foundation for future statistical analyses and the conclusions drawn from them. Online chess websites that enable online play. Each Internet portal for chess-playing mentioned in Section 2 offers a dedicated application for mobile devices. According to the International Chess Federation (FIDE), chess apps have been installed 1 billion times on smartphones. Widespread availability and easy use brought about a surge in the number of players. Since the outbreak of the pandemic, the number of games played online each day has increased by around 50%, to around 6.5 million. The numbers of games played have, therefore, the potential to be analysed globally, including for purposes other than pure chess analysis.

The next step is to determine the format in which the information is to be stored. Standardisation will make using the database, analysing and interpreting the information stored therein much easier.

The next step in creating the database will be to evaluate the individual moves of the players in order to assess the quality of the decisions made. The specifically designed algorithm will assess each move, both white and black, and simultaneously assign a numeric value to them. For this purpose, it will use already analysed games stored in the database or its own analysis based on an artificial intelligence algorithm (chess engines).

### 4.2. Stage 2—Segmentation of Groups of Chess Players

Stage 2 will involve the development of theoretical algorithms to segment the individual players, taking into account the games they have played (putting players into groups according to their skill level). It is necessary to identify such players and lists of chess games that prevent the evaluation of the individual players’ playing competence and allow focusing on the quality of the decisions made. Such an inference will be possible if games of players at the same level of play are accepted for analysis because the game of chess brings together different players with varying skills and experience. The assumption for selecting players for analysis is to match them in terms of playing strength. For this purpose, player rankings will be reviewed. The best way is to use the Elo rating because each player is assigned a chess category and an Elo rating. Where no information on a player’s Elo ranking is available, national chess categories will be used. Chess titles and categories are an exponent of the maximum sports score achieved by a chess player. In direct play, we distinguish: female and male titles and categories, international titles (categories) and national central and national district categories.

The higher the level of players, the lower the number of errors, and the better the quality of the games played. Hence, the statistical data will be of a completely different nature. Accordingly, critical statistical levels will be adopted for each ranking bracket, based on which the error in the move will be determined.

Segmentation of groups of chess players aims to in order to match opponents properly according to the criterion of playing strength. Depending on the area involved in the decision-making process, measures of decision quality tend to vary. The full basic scope of the definition of a measure includes [65] the name of the measure, the purpose, the frequency, the way it is measured and calculated (or estimated), the source(s) of data, the responsibility for values and measures, the target value. The ideal measure should be quantitative or value-based and, above all, objective. The measure should also be understandable, transparent and easily accessible. The use of the Elo ranking classification and the analysis of moves made during a chess game, accompanied by time information, seems to meet these criteria. One of the key tools developed for the study of chess is the Elo chess scale [66] which can serve as an important instrument for studying individual differences between players. The Elo measure is frequently employed in research e.g., [63,64,67], which supports its usefulness. Together with information on the player’s background (region/country), it forms an invaluable source of knowledge regarding behaviour/personality/decision-making. The Elo classification is a standard measure of a player’s chess skills and is determined by the results of matches recognised by FIDE (the acronym FIDE comes from the French name Fédération Internationale des Échecs). FIDE stands for the World Chess Federation and collects information on the official Elo rankings of chess players. FIDE data includes the players’ ELO ratings in standard chess, rapid chess and blitz chess. Standard, rapid and blitz chess differ only in the time allocated to each player per match. With the data collected by FIDE, it is possible to assess how the decision-making process and its outcome are affected by the rigour of time constraints.

Players will be assessed using the Elo ranking described above, which measures a player’s playing strength. The higher the rank, the greater the playing strength. This ranking is subject to change, which means that it may also decrease. It constantly fluctuates—depending on the player’s strength—according to one’s progress or performance. Depending on the pace (game time per player) of the tournament, we can distinguish three rankings:

- Standard—for classic chess (pace ≥ 60’/90’/120′ per player depending on player’s rating)
- Rapid—for rapid chess (pace ≥ 10′ per player)
- Blitz—for rapid chess (pace ≤ 10′ per player)
- For example, if you only play in rapid chess tournaments, you have only the FIDE Rapid ranking.

The ranking list is published on the first day of each month. If a player participated in FIDE-rated tournaments in the preceding month, the ranking would reflect any changes. This approach means that the current playing strength of an individual player can be determined very quickly. Online games update the players’ rankings after every game played. This allows sorting the players by their respective strength of play, taking into account the current shape of each player.

Online chess portals use the Elo algorithm to determine the strength of their players. The *R* ranking score achieved in a rated competition is calculated using the formula (1):

(1) $$R_{u}=R_{s}+\Delta R,$$

where: *R_s_*—the value calculated according to the formula (2):

(2) $$R_{s}=\frac{1}{n+1}\sum_{i=1}^{n+1}R_{i},$$

- $\sum_{i=1}^{n+1}R_{i}$ is the sum of the rankings of all players participating in the tournament. In the case of team and Swiss competitions, it means the sum of the rankings of the rated player and his/her opponents
- n is the number of games played (in practice, it is also the number of opponents of the rated player),
- and ΔR is calculated using the formula (3):
  (3) $$R_{s\;}=\frac{1}{n+1}\sum_{i=1}^{n+1}R_{i},\;\Delta R=\frac{400}{n+1}\left(W-P\right)$$
  where: *W*—wins, *P*—losers.
- The Δ*R* values depend on the score achieved in the competition (*W* − *P*) and the number of games played.

The player rankings also take into account the development coefficient (K), which can be crucial in selecting individual players for future analyses. When selecting study subjects, it is important to remember to pick candidates carefully for the study sample. To this end, it is critical to be mindful of the shape of individual players and the factors influencing the algorithm for calculating player rankings.

### 4.3. Stage 3—Development of Selection Assumption

The objective of Stage 3 will be to determine the size of research samples that allow conclusions to be drawn for entire social groups. The use of research sample selection algorithms allows us to assume that the analysed data will be scalable to the entire study population. The minimum sample size will be determined based on: the estimated prevalence size, maximum estimation error, significance level, and size of the general population (for finite population).

This stage will also see the validation of the acquired data, which aims to verify the data. This will be done in order to detect any incomplete data, values of variables that are outside the acceptable range and combinations of variable values that have been introduced by mistake. Furthermore, variables will be categorised by types, the identification of which is necessary to perform statistical analyses (identification of dependent and independent variables).

Since the reliability of the results is affected, among other things, by the size of the research sample, the process of selecting the sample size will be an essential aspect of the study. The most important factors that have an impact on the accuracy of the representative method are the structure of the community, the sampling scheme used and the sample size. The minimum sample size can be determined based on the parameters:

- the estimated size of the prevalence, i.e., the proportion of individuals meeting the specified characteristic;
- maximum estimation error;
- significance level, which is interpreted as the probability that an estimation error of a given maximum value is made;
- the size of the general population (for a finite population).
- For a finite population, the formula for minimum sample size presents itself as follows (formula (4)):

(4) $$N_{min}=\frac{P\left(1-P\right)}{\frac{e^{2}}{z^{2}}+\frac{P\left(1-P\right)}{N}},$$

whereas for an infinite population (formula (5)):

(5) $$N_{min}=\frac{z^{2}P\left(1-P\right)}{e^{2}},$$

where:

- *P*—estimated prevalence size,
- *z*—value resulting from the assumed significance level (α), calculated using the normal distribution function
- *N*—size of the general population (in the case of a finite population),
- *e*—maximum estimation error.

The sample size that ensures obtaining a predetermined precision of the interval estimate of the mean is expressed by the quantity (formula (6)):

(6) $$n=\frac{z_{\alpha }^{2}\delta^{2}}{e^{2}},$$

Note, however, that the application of this formula requires the normality of the distribution of the tested variable and a constant and known σ2 variance. If the general population follows a normal distribution with an unknown variance σ2, the minimum sample size can be determined using the so-called “Stein two-stage method”. The sample size is then calculated based on the formula (7):

(7) $$n=\frac{t_{\alpha ,n_{0}-1^{s^{2}}}}{e^{2}},$$

Table 1 provides an illustrative breakdown for each European country, showing the required minimum research sample (the number of people needed for the research to be carried out) and the number of chess players registered with FIDE (having an Elo ranking).

The overview provided indicates that in the course of the study, somewhere between 7744 and 9604 players should be surveyed for a maximum error of 1%, a confidence level of 95% and a prevalence size of 0.5. For 15 countries, these requirements are met only for the players listed in the FIDE registers who have complied with the specified requirements. However, it should be noted that the number of online players is much larger than the number of players registered with FIDE. It is estimated that around 1 billion people on Earth know the chess moves, which represents a gigantic potential to carry out global social geospatial analyses, with very little investment, yet based on reliable and relevant statistical results. In contrast, Table 2 shows the countries for which the maximum error was calculated for the number of registered FIDE players. The results show unequivocally, considering only players registered with the FIDE federation, that the maximum error would exceed 5% only for Liechtenstein and Monaco (statistical inference would be inaccurate above this figure).

### 4.4. Stage 4—Inference Statistical

The most important Stage 4 consists of statistical inference. Correlation matrices of observations will be created as a first step. The detailed analysis of the information will concern the evaluation of the relationship between the data: nationality (geographical location), game results, gender, quality of decisions made, characteristics of the choices made by each nationality, time of day against the result (optimal time for cause-and-effect thinking). Given the vast number of observations, proprietary algorithms will be created to facilitate the interpretation of information. The main task will be to identify correlations between the quality of decisions made and the geographical location (origin) of the players. A derivative of the conducted study will include analyses related to the possible influence of personality trait variables (gender, age) on decision-making ability.

Equipped with the assessment of the quality of decision-making in a chess game (based on the chess engine) and the methodology for selecting research samples (based on the Elo ranking and the size of the sample), one can proceed to geospatial analyses, drawing on correlation analysis. It is generally accepted that empirical studies can fall into one of two categories: correlational or experimental studies. In this project, the focus of the analysis will be placed on observing the resulting correlations. In the correlational study, the researcher does not interact with any of the variables, merely recording them and observing the relationships (correlations) between certain subsets of the variables. Data from correlational studies can be interpreted only in causal terms. First, the variables are going to be differentiated and grouped into dependent and independent variables. Discovering the dependencies between variables is the primary objective of any scientific study. Regardless of what type they are, two or more variables are related if the values of these variables are distributed in a specific, systematic way in the measured sample. The statistical significance of the result, i.e., determining its representativeness for the entire population under study, plays a vital role in the process of statistical inference. The decision as to what level of significance we are inclined to consider truly significant is always taken arbitrarily. As far as a sample of a specified size is concerned, the greater the strength of the relationship that exists between the variables, the more significant the relationship is.

In the event of a large number of observations, there will be a corresponding presence of all possible combinations of different values of the individual variables. The probability of an accidental occurrence of a combination indicating a strong correlation in the measurement done for a small set of data is relatively high. In the present case, such a risk will be minimised. From the statistical point of view, small effects can only be detected with large-size samples. The assumed procedure to conduct such an assessment is to examine the differentiation (variability) of the values of the measured variables and then to calculate what proportion of this generally available variability can be attributed to the fact that the variability is common to two or more of the variables under study. General characteristics of the variables, such as mean, median, standard deviation, and information about the distribution of the variables will serve as the starting point. All data will be subjected to statistical tests based primarily on the normal distribution of the variables.

### 4.5. Chess Information—Game, Move, Result

As previously mentioned, the database will be formed on the basis of completed chess games. In order to be able to use the assessment of moves made in a chess game for social geospatial analysis, it is necessary to analyse the full process of how this assessment functions. Assessing a single move for both the white and black pieces can be a measure of the quality of the decisions made. The analysis of the players’ individual moves will be carried out using a selected chess engine. The information about played chess games accumulated in chess databases will serve as the main source of data.

A chess engine is software that is used to analyse chess positions and generate the moves it deems best. Some of the most powerful chess engines include: Stockfish, (https://www.computerworld.pl/porada/Cel-zaklety-w-miernikach,293796.html accessed on 10 August 2022) Leela (v0.28.2., LCZero project, international community, 2021) and AlphaZero(first version, DeepMind, London, UK, 2017). Stockfish is one of the world’s finest chess engines. It is used to assess whether a move made is perfect, good, weak or bad. The classification of the move is determined upon the evaluation of the position. This engine is able to determine which player is winning. If a player makes a move that puts them in a position that the engine considers a losing one, then that move will be considered a poor move or an error. A good move is a move that helps positively contribute to the position but is not the best move according to the engine. A perfect move is one that matches the engine’s proposal in a given position. AlphaZero is a unique chess engine developed by Google. AlphaZero uses Deep Learning. AlphaZero played millions of games on its own, slowly improving until it reached a point where AlphaZero was able to beat Stockfish. Leela Chess Zero is an open-source implementation of AlphaZero. Leela used a similar process to that used by AlphaZero. Auto-play learning was used to enhance the game.

**Figure 3:**
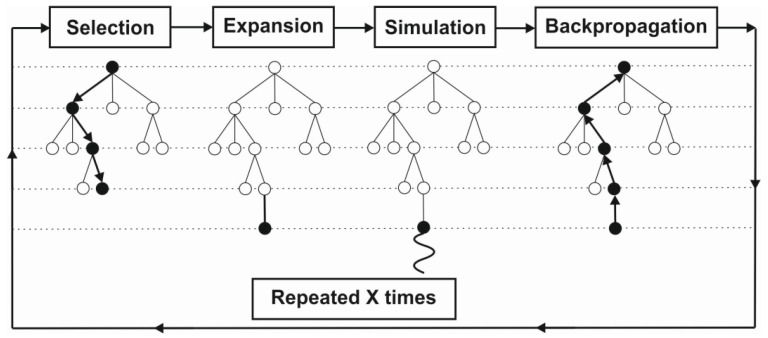
Example of a Monte Carlo tree search. Source: Own study based on [69].

We can distinguish two trends in the development of chess engines. The first is classic evaluation, where the rating comes from an algorithm that is manually created by chess experts. The second approach is the use of Deep Learning. It uses a deep neural network, which is a network with multiple layers between the input and output layers. In both of these solutions, a form of tree search is needed to efficiently search and evaluate the moves. Tree search will use a common data tree structure. A data tree is a collection of nodes that branch off from a parent node. There are several ways to search a tree. Among the most important, we can include Alpha-Beta pruning and Monte Carlo tree searching. The Stockfish chess engine uses a man-made algorithm and techniques such as Alpha-Beta pruning, while AlphaZero employs Monte Carlo Tree Search (Monte Carlo Tree Search—Figure 3) and a deep neural network [68].

**Figure 4:**
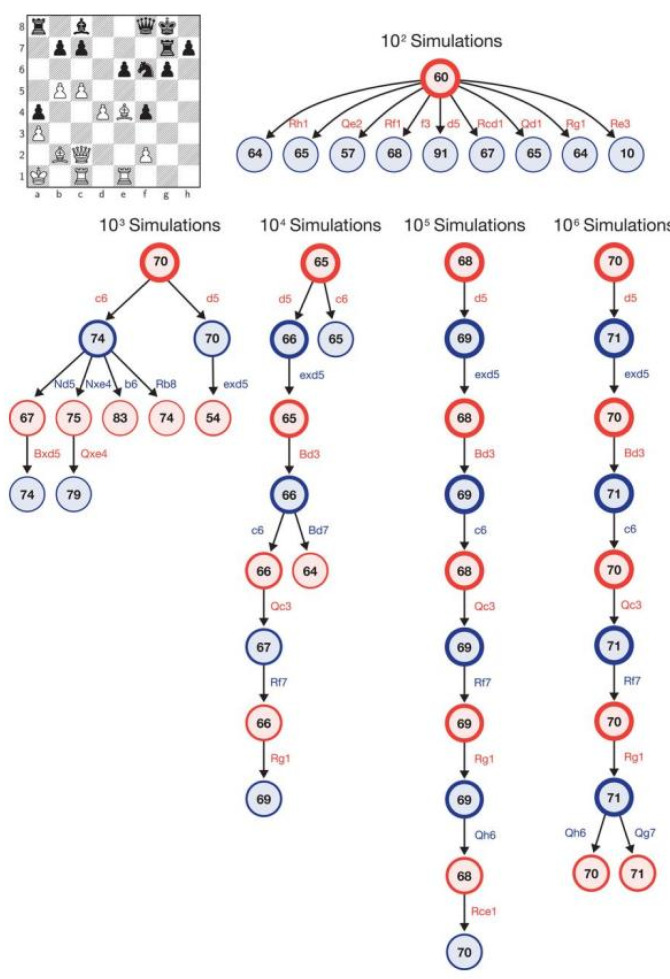
Monte Carlo Tree Search used for a given chess position. This figure shows a Monte Carlo Tree Search being used for a given chess position. Each simulation summary shows the top 10 most visited states and the estimated value for a move that was simulated. Source: [70].

The positions on the board constitute the input data for a deep neural network. The output is a vector of move probabilities. The move probability is given a value based on the expected outcome of the game. Figure 4 shows how AlphaZero would use the MCTS algorithm for a given position. In the end, each simulation is associated with a final outcome in the variant: win takes + 1, loss takes −1, and a draw takes the value of 0.

Authors should discuss the results and how they can be interpreted from the perspective of previous studies and the working hypotheses. The findings and their implications should be discussed in the broadest context possible. Future research directions may also be highlighted.

Below is an example of the structure and format for assessing the progress of a chess game. The standard form of chess game notation is shown in Table 3.

The moves are noted down in sequence immediately after each move. On the left are the moves of the white player, and on the right are those of the black player. This structure allows making an assessment after each move of each side.

**Figure 5:**
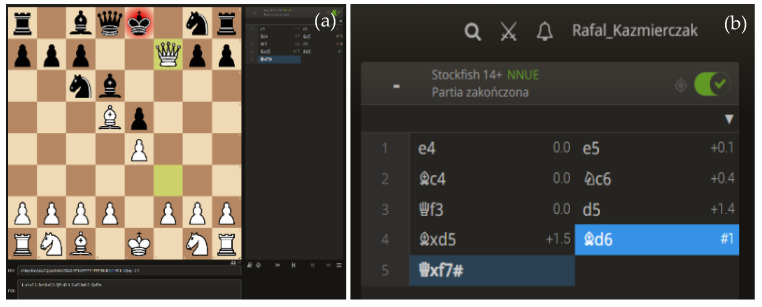
Example of a chess game with move analysis performed by the chess engine: (**a**) current move, (**b**) the record of the game. Source: own study.

The above figures illustrate a sample analysis of a game on the lichess.org web portal. Figure 5a shows the current move, and Figure 5b shows the record of the game. In the second figure, we see the record of the white (left) and black (right) side moves. In addition, the position is continuously analysed and assessed using an artificial intelligence algorithm (chess engine). For example, after the first move—e4, there is a rating of 0.0 right next to the move description. A similar rating is given after black’s response. In this case, black’s answer was e5, which was given a rating of + 0.1. In the case of the chess engine assessment, the 0.0 rating informs us about equal chances for both sides. This means that the moves performed on both sides are the best. The computer makes its assessment from white’s perspective, which is why the notation adds plus or minus before the displayed number. In one of black’s next moves (#3), there was the move d5, which was rated by the chess engine as a weak move, with the rating of + 1.4. This means that white, with its best response, will achieve a positional advantage. Black made another mistake in the next move and was checkmated on move 5, effectively meaning that white won the game. With the ability to assign a rating for each move and to record the time the player took to think about it, we can perform analyses on the quality of the decisions made. A characteristic feature of the model adopted is that, depending on the strength of the players’ game, the computer evaluation of the position will vary considerably. The higher the level of the players, the fewer errors, which, at the same time, will translate into smaller values for the position assessments. Such a structure makes it possible to carry out highly detailed analyses. In addition to the description of each chess game, there is obligatory information, such as the name of the player and his rating and the date the game was played.

### 4.6. Configuration of Possible Combinations of Information

**Figure 6:**
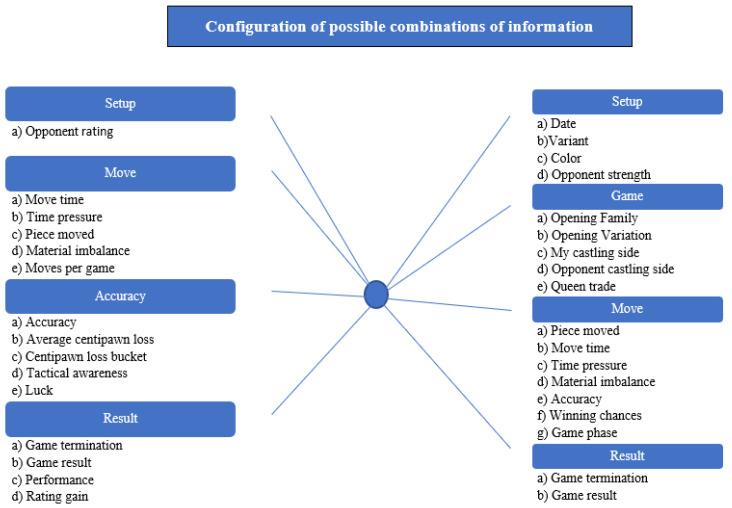
Overview of possible combinations of data from completed chess games. Source: own study.

It should be noted that the number of possible combinations of datasets derived from chess games played is 528. This is shown in Figure 6.

It is important to remember that each combination provides opportunities for different inferences. The number of combinations increases depending on the use of the filter. The filtering options are shown in Figure 6. We have a choice of different options in each group: for Player—8 possibilities, for Game—38 possibilities, for Move—47 possibilities, for Result—9 possibilities. This amounts to a total of 102 possibilities of basic filtering, which, combined with the number of all possible combinations of data from completed chess games (528), gives a multidimensional possibility of creating various compilations of data and drawing conclusions from them. As a result, it is possible to generate at least 53,856 datasets—thus, analyzing the obtained information is possible in many different ways.

### 4.7. Geostatistics

In order to make the interpretation of the acquired data easier, it is planned to employ geospatial tools. The analysis of spatial relationships will facilitate drawing conclusions regarding the spatial distribution of the obtained results and make the interpretation easier to understand. These tools help to identify, quantify and spatially visualise trends in the data. The use of the following tools is planned:

- calculation of density—this tool creates a density map from point objects by appropriately distributing the known values expressing the given phenomenon (represented by point attributes) on the map surface. The result is a layer of areas representing density.
- find hot spot locations—the tool allows the user to determine whether statistically significant clusters exist in the spatial relationships of their data.
- find point clusters—the tool finds clusters of point objects among the surrounding noise based on their spatial distribution.
- classification and regression based on decision tree sets—the tool models and generates predictions using a customised algorithm of random decision tree sets (forests), which provides an example of a supervised machine learning method.
- generalized linear regression—the tool is used to generate forecasts or model the dependent variable in the context of its relationship with a set of explanatory variables. This tool can be used to fit continuous (OLS), binary (logistic) and count (Poisson) models.

## 5. Configuration of Possible Combination of Information—Examples

As previously mentioned, there are a number of possibilities for making compilations of information from completed chess games. Virtually any of the elements mentioned can be used for social geospatial analyses. Below are some specific examples showing the potential for analysis, illustrated with drawings from the lichess.org platform. Due to the limited size of the article, most of the drawings are included in the supplementary materials (Figures S1–S12), and only selected ones have been incorporated into the text.

Based on a large number of observations, it is possible to draw conclusions of a global character. Naturally, due to the varied level of chess performance, these conclusions will be different for each group of players under study, but it is possible to determine the risk propensity of individual chess players by observing their opening choices. We have different types of chess openings: open, semi-open and closed. Each of these has different characteristics and requires different personality predispositions. It is assumed that people who like lengthy maneuvers and the defensive nature of the game are more inclined to slower, closed variants. Those with more temperament prefer dynamic openings. They very often reach for gambits, i.e., a substantial sacrifice in exchange for a dynamic development on the chessboard. Hence, a popular saying among chess coaches is: ‘Show me your chess games, and I will tell you who you are’, which, in the age of access to numerous databases, can be translated into the language of statistics. Through the use of various filters, it can be verified whether the above saying can be applied to individual nationalities, for example. The outline of the general principles of such analyses presented in this article is illustrated using the sample statistics of a selected player.

**Figure 7:**
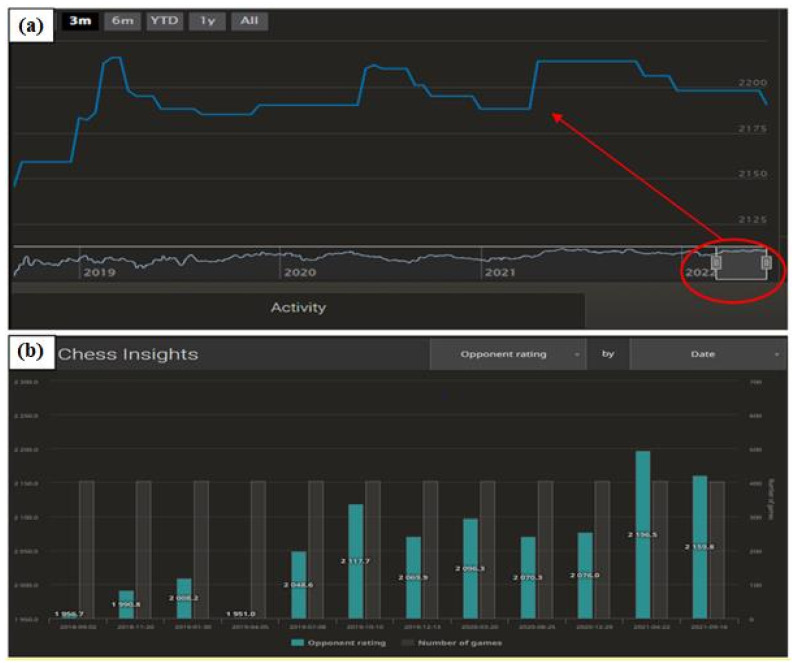
The results of the attained chess ranking on the Lichess platform (**a**) and the correlation of the average ranking of the opponents in different time intervals (**b**). Source: lichess.org.

Figure 7 shows a selected attained chess ranking of a player, which covers the period from 2 September 2018 to 12 July 2022. During this period, 4888 chess games played were used to determine the ranking. One important yet natural aspect is the trend of the graph in the first period. For each player, it takes an upward pattern until the strength of play stabilises. Filters set on the players’ accounts lead to opponents being selected in the first period according to playing strength, i.e., the player’s chess ranking. In the initial phase, the new player has a hidden playing strength, and his/her ranking does not correspond to the skills possessed. This causes the player to mostly win and increase the ranking very quickly. The ranking changes after each game played; hence, after a few dozen duels have been played, the ranking stabilises and starts to reflect the true playing strength of the player. This is crucial for the selection of the research sample. Figure 7b shows the changes in the average playing strength of the opponents over the different periods. Naturally, the figure correlates with Figure 7a—when the playing strength increased, so did the level of opponents.

Numerous conclusions regarding the impact of time pressure and length of deliberation on the quality of decisions can be drawn based on breakdowns showing the length of deliberation for a single move. Such analyses can be performed for an individual player and for a selected group of players (filtered using a preselected key). For a three-minute game, the breakdown of the average time spent on a move does not depend too much on the strength of the opponent but only on the short time available to play the entire game. In this case, a change in deliberation time by one-tenth of a second is statistically significant (Figure S1). Again, confirmation of the assumption that the best research sample consists of opponents with the same playing strength can be noted. In this case, the longest time spent per move is 3.8 s. This is attributed mainly to the longer equilibrium and the necessity to solve a greater number of problems and make more decisions. When it comes to weaker players or much stronger players, it is easier to make moves since the predicted outcome is known beforehand.

From a logical point of view, at a lower level of playing strength, the more possibilities on the chessboard, the more time the player should spend on analysis, as there are more options to analyse. Of course, this depends on the complexity of the position, but when analysing the average time spent on the move of a given piece, it is evident that the time spent to move the Queen is the highest (Figure S1b). This can be linked to the fact that Queen is the strongest piece on the chessboard and has the most possibilities to make a move along straight lines and diagonal lines. Conversely, King and Pawn moves required the least amount of time. The reason for this is the limited possibilities of the individual pieces, which can only be used to make the simplest moves.

Based on statistics, stereotypes can be challenged (Figure S2). There is an expression “reflexes of a chess player”, which refers to slow and phlegmatic people. However, nowadays, it has little in common with reality. One can observe a phenomenon quite the opposite of this stereotypical approach. Chess players’ reflexes and swiftness of logical response are impressive, even in players presenting a semi-amateur level. In the example shown, 44,620 moves were made in 0.34 s. Before the move was made, in a prevalence of a second, the position had been assessed and analysed and, based on this, the most logical move for the situation was selected according to the player’s judgement. Considering the time in which this was done, the result is impressive. This stems from the simple fact that everyone plays to win. To achieve victory, they have to make rational decisions, the best possible ones given their knowledge. Hence, regardless of the level of the player, they always strive to make the best possible choice.

**Figure 8:**
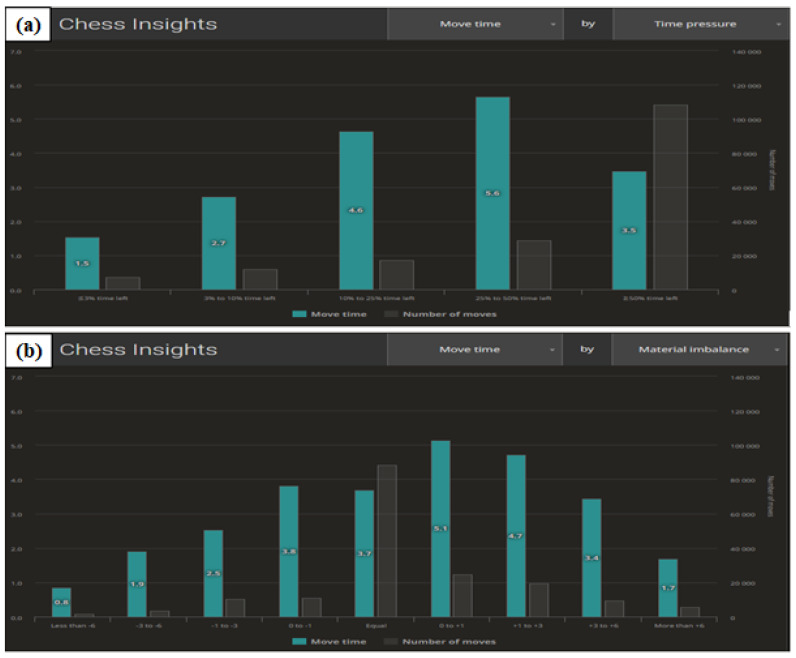
The effect of the pressure of elapsing time on the length of deliberation on a move (**a**) and the correlation of the average time spent on a move with the evaluation of the chess position (**b**). Source: lichess.org.

The next example proves that it is possible to monitor the effect of time pressure on decisions across a large number of participants. Figure 8a shows the correlation between average time per move and the pressure of elapsing time.

The figure shows that the greater the time pressure, the less time is spent on deliberation. This juxtaposition should be correlated with Figure S3 showing the accuracy of decision-making. Figure 8b illustrates the correlation of the time required for deliberation in a situation of material inequality on the chessboard. Inequality is understood as an advantageous position for one of the players. The advantage is assessed by artificial intelligence algorithms. When it comes to weaker players, it is very often equated with having an advantage in one player’s number of pieces. The higher the level of the players, the more important the role of the balance and positioning of the pieces, rather than their number. It can be seen in the figure that a nearly equal position requires more thought.

Figure S4 shows the correlation between the average deliberation time vs. the accuracy of the move suggested by the Stockfish chess engine. On this basis, it is possible to determine the ability to exploit emerging opportunities but also the ability to see the game through to the end without unnecessary distraction or additional risk.

Another crucial piece of information is the correlation between time pressure and decision-making (Figure S5). We interpret time pressure as the time remaining in the game, i.e., accounting for increment (100% = full clock, 0% = flagging). In both cases under analysis, it can be seen that the quality of decisions made is weaker in the middle game, where there is the greatest complexity of positions. The fact is that the greatest amount of time is spent on the middle game during a match. The first stage of the game, i.e., the opening, is usually known to the players, hence they spend less time deliberating. The middle game is the most challenging due to the complexity of the position, and the lower values for endings appear primarily due to time pressure, which reduces the time available for deliberation. From the point of view of analysing the decisions made, this can prove to be useful information about the way a particular game ends, such as whether one fights to the very end, regardless of the chances of winning. Or does one play to finish with a checkmate? Statistics can show some generalised trends (Figure S6).

**Figure 9:**
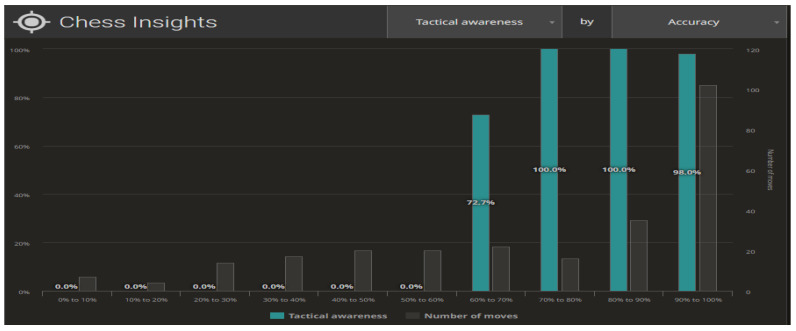
Statistics on taking advantage of opponent’s mistakes. Source: lichess.org.

**Figure 10:**
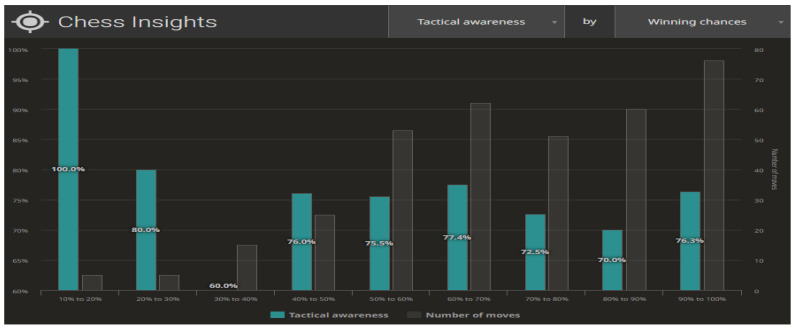
Statistics on making the most of opportunities to win. Source: lichess.org.

The different configurations of the datasets from the completed games allow multiple analyses to be conducted on different public groups. Figure 9 and Figure 10 illustrate the compilations for the Centipawn loss bucket (Centipawns lost by each move, according to Stockfish evaluation). Both in life and in chess, we are able to distinguish between two basic elements of planning—long-term and short-term planning. In the context of chess, long-term planning usually involves strategic (positional) play and short-term planning means tactical action. Tactics in chess refer to the combinations appearing on the chessboard that lead to material advantage. Typically, tactics involve multiple possibilities and a large number of variants, yet spanning 3–5 moves ahead. The level of skill in calculating tactical variants can provide information about a player in terms of their skill and accuracy in analysing a problem. As far as chess is concerned, we can contrast Tactical awareness (How often you take advantage of your opponent’s mistakes) with Accuracy (How accurate your moves are, based on Stockfish evaluation) (Figure 9). The second aspect being analysed is the ability to take advantage of the opportunities created (Chances to win a position, based on Stockfish evaluation. A.k.a. Win%) (Figure 10).

When analysing a single game using the chess engine, we can see the phases of a chess game separately, and each individual move is evaluated. The turning point in the game is clearly visible in the analysis. Based on the assessment of the accuracy of the performed moves, one can look for reasons for poor or very good decisions. In the case of a single player, this can be used to improve individual skills, not only those concerning the game of chess. For example, it can provide information on how a person copes with stress. For collective analyses, generalised conclusions can be drawn from individual research samples (e.g., children and adults, men and women, nationalities, etc.).

## 6. Summary and Conclusions

Future large-scale studies of the geographical variation of personality traits employing the developed methodology of using chess games have the potential for a number of applications. The findings in this area can be used wherever cross-sectional social analyses are required in the context of personality traits (decision-making) to better understand their geographical origins. In turn, the geographical distribution of these traits is associated with a number of important social, health, political as well as economic implications. When combined with geolocation, the analysis of correlation coefficients will allow for clear visualisation (in the form of a thematic map). If positively confirmed, the potential of the analysis of chess games creates an unprecedented and unique opportunity for social analyses regarding the relationship between data on nationality (geographical location) and the quality of decision-making and characteristics of the choices made by different nations.

The idea of using the game of chess for geographical analysis is also based on its popularity. As a matter of fact, chess is for everyone. Chess exercises causal analytical thinking skills regardless of the level of play. This leads us to assume that chess can become a truly universal test of logical thinking competence. Since chess is a deterministic game of individuals, and chess games are accurately documented and digitally available (every game streamed or played over the Internet is recorded), chess data are a potential source of information that can be used to study regional differences in traits in strategic behaviour, as well as latent individual traits (personality traits—decision-making ability).

Played chess games can serve as a great analytical tool, and not just in terms of mathematics or computer science. Analyses of the results of other sports or international tests are carried out using selected social groups or groups of professionals. This is the case, for example, with intelligence tests or sports tournaments of championship rank. Such a situation means that we have detailed statistical data for a selected, small group of people only. In the case of chess, the fact that players (both amateurs and professionals) play and train online makes it possible to conduct analyses across very wide and diverse study groups. Access to information on the entire, wide group of people makes it possible to analyse the factors influencing the course of a chess game in a simple and easy way, and extremely large study groups allow for correct statistical inference.

It should be emphasised that the ability to make decisions is of crucial significance in the game of chess in terms of the achieved results—and so they can provide an invaluable source of information in this respect. The gigantic popularity of the game of chess and the corresponding huge database covering the entire world makes it possible to analyse played games not only in terms of chess quality but also for the purposes of various statistical analyses related to player attributes. The rules of chess and analytical advances in modern computer science make it possible to treat the results of chess games as an international test of the quality of decision-making processes in relation to the players’ backgrounds (geographical location). This approach is innovative for this type of analysis.

The findings in this area can be used wherever cross-sectional social analyses are required in the context of personality traits (decision-making) to better understand their geographical origins. In turn, the geographical distribution of these traits is associated with a number of important social, health, political as well as economic implications. When combined with geolocation, the analysis of correlation coefficients will allow for, among other things, the identification of the needs and incidence of risk or particular social groups’ vulnerability to stress. It will also allow for early prevention and enable further development from an early age. The results can be applied when introducing kinesiology education and its use in teaching preschool and early school-age children [71]. An individual analysis of a single player may be an indication for work on improving the functioning of the mind (improving memory, concentration, eye-hand coordination, articulation, reading, counting, memorising numbers and writing). The results of the analyses may prove useful when working on the psychological rehabilitation of people with chronic diseases. The main idea is to use the game to engage the patient emotionally and to encourage any kind of activity. A considerable number of observations may allow us to juxtapose the results obtained with medical and laboratory research focusing on the medical aspects of the functioning of the human mind. The results of the conducted research may also contribute to the even greater popularisation of the game of chess and its use in the educational process to an even greater extent. This is all the more important because it has been confirmed that chess has a positive influence on educational results. Children who play chess exhibit long-term memory organisation, in-depth problem analysis and the ability to cope with problem-solving [72,73].

It should be noted that there are three main areas of risks related to the implementation of the presented methodology. The first risk is technical issues related to supplementing the database using webscraping algorithms. The second risk is the quality of the information in the database (e.g., reliability of player information, gaps in the database, etc.). The third group of risks is related to the correct statistical inference. For a large number of observations, there will be a large number of possible combinations of different values of particular variables. The chance a combination indicating a strong correlation will occur in the measurement is relatively high. In the present case, such a risk will be minimised. From a statistical point of view, small effects can only be detected when using large-size samples. The assumed procedure for such an assessment is to examine the differentiation (variability) of the values of the measured variables and then to calculate what proportion of this generally available variability can be attributed to the fact that the variability is common to two or more variables under study.

## Figures and Tables

**Table 1:** Population in each European country and minimum research sample size.

Country	Population [Number of Persons]	Number of Chess Players Registered with FIDE [Persons]	Minimum Research Sample [Persons] Prevalence Size: 0.5, Maximum Error: 1%, Confidence Level: 95%	Minimum Research Sample [Persons]. Prevalence Size: 0.5, Maximum Error: 2%, Confidence Level: 95%
Russia	146,000,000	107,021	9604	2401
Turkey	84,680,000	46,881	9603	2401
Germany	83,160,000	42,181	9603	2401
France	67,630,000	80,037	9602	2401
United Kingdom	67,530,000	12,698	9602	2401
Italy	59,240,000	27,778	9602	2401
Spain	47,400,000	62,993	9602	2401
Ukraine	41,000,000	16,497	9601	2401
Poland	37,840,000	34,488	9601	2401
Romania	19,200,000	11,146	9599	2401
Netherlands	17,480,000	9011	9598	2401
Belgium	11,550,000	7668	9596	2400
Greece	10,680,000	25,604	9595	2400
Czechia	10,500,000	14,898	9595	2400
Sweden	10,380,000	7289	9595	2400
Portugal	10,300,000	7180	9595	2400
Hungary	9,730,000	11,686	9594	2400
Belarus	9,250,000	2831	9594	2400
Austria	8,930,000	8020	9593	2400
Switzerland	8,670,000	5036	9593	2400
Serbia	6,870,000	11,105	9590	2400
Bulgaria	6840,000	5303	9590	2400
Denmark	5,840,000	7492	9588	2400
Finland	5,550,000	1915	9587	2400
Slovakia	5,460,000	8386	9587	2400
Norway	5,430,000	7340	9587	2400
Ireland	5,010,000	2060	9585	2400
Croatia	4,040,000	10,355	9581	2399
Bosnia and Herzegovina	3,460,000	3184	9577	2399
Albania	2,830,000	786	9571	2399
Lithuania	2,800,000	5302	9571	2399
Moldova	2,600,000	1237	9568	2399
Slovenia	2,110,000	5642	9560	2398
Republic of North Macedonia	2,070,000	1286	9559	2398
Latvia	1,890,000	3796	9555	2398
Kosovo	1,770,000	780	9552	2398
Estonia	1,330,000	2108	9535	2397
Cyprus	900,000	1144	9502	2395
Luxembourg	630,000	725	9459	2392
Montenegro	620,000	1178	9457	2392
Malta	520,000	414	9429	2390
Iceland	370,000	2446	9361	2385
Faroe Islands	50,000	453	8056	2291
Liechtenstein	40,000	31	7744	2265
Monaco	40,000	207	7744	2265

Source: Own study based on (https://ratings.fide.com/, https://pl.tradingeconomics.com/country-list/population?continent = europe (accessed on 10 August 2022)).

**Table 2:** Maximum error percentage for the number of players registered with FIDE in each European country.

Country	Population [Number of Persons]	Number of Players Registered in FIDE [Persons]	Maximum Error
Finland	5,550,000	1915	2%
Ireland	5,010,000	2060	2%
Albania	2,830,000	786	3%
Kosovo	1,770,000	780	4%
Estonia	1,330,000	2108	2%
Cyprus	900,000	1144	3%
Luxembourg	630,000	725	4%
Montenegro	620,000	1178	3%
Malta	520,000	414	5%
Faroe Islands	50,000	453	5%
Liechtenstein	40,000	31	18%
Monaco	40,000	207	7%

Source: own study.

**Table 3:** Classical format of a chess game notation.

Move	White	Black
1.	e4	e5
2.	Nf3	Nc6
3.	Bc4	Bc5

Where: Knight (N), Bishop (B), Rook (R), Queen (Q), King (K).

## Data Availability

Not applicable.

## Supplementary Materials

The following supporting information can be downloaded at: https://www.mdpi.com/article/10.3390/ijerph191912353/s1, Figure S1. Correlation of the time spent per move taking into account the strength of the opponents (a) and the average time spent making a move with an individual piece (b); Figure S2. Statistics concerning the number of moves in each time slot; Figure S3. The analysis of a single game using the chess engine (a) and the statistical analysis that takes into account the time spent per move (b); Figure S4. Evaluation of the quality of the decisions made, with regard to the average time per move (a), and evaluation of the chances of winning in relation to the time spent per move (b); Figure S5. Correlation of time pressure and accuracy of decision making (against chess engine evaluation) (a) and overview showing accuracy with time pressure taken into account (b); Figure S6. Game termination type statistics (a) and game result statistics (b); Figure S7. Correlations between Centipawn loss bucket and Move time; Figure S8. Correlations between Centipawn loss bucket and time pressure; Figure S9. Correlations between Centipawn loss bucket and Material imbalance; Figure S10. Correlations between Centipawn loss bucket and Accuracy; Figure S11. Correlations between Centipawn loss bucket and Winning chances; Figure S12. Correlations between Centipawn loss bucket and Game phase.

## References

[1] Chen H., Lai K., He L., Yu R. (2020). Where You Are Is Who You Are? The Geographical Account of Psychological Phenomena. Front. Psychol..

[2] Rentfrow P.J. (2020). Geographical psychology. Curr. Opin. Psychol..

[3] Rentfrow P.J., Carducci B.J., Nave C.S., Mio J.S., Riggio R.E. (2020). Personality and Geography. The Wiley Encyclopedia of Personality and Individual Differences: Clinical, Applied, and Cross-Cultural Research.

[4] McCrae R.R., Costa P.T. (2003). Personality in Adulthood: A Five-Factor Theory Perspective.

[5] Allik J., McCrae R.R. (2004). Toward a geography of personality traits: Patterns of profiles across 36 cultures. J. Cross-Cult. Psychol..

[6] Rentfrow P.J., Jokela M., Lamb M.E. (2015). Regional personality differences in Great Britain. PLoS ONE.

[7] Wood D., Rogers K.H. (2011). Regional differences in personality exist, but how do we get to them? The case of conscientiousness. Am. Psychol..

[8] De Montesquieu C. (1989). Montesquieu: The Spirit of the Laws.

[9] Pennebaker J.W., Rimé B., Blankenship V.E. (1996). Stereotypes of emotional expressiveness of Northerners and Southerners: A cross-cultural test of Montesquieu’s hypotheses. J. Personal. Soc. Psychol..

[10] Linssen H., Hagendoorn L. (1994). Social and geographical factors in the explanation of the content of European nationality stereotypes. Br. J. Soc. Psychol..

[11] McCrae R.R., Terracciano A., Realo A., Allik J. (2007). Climatic warmth and national wealth: Some culture-level determinants of national character stereotypes. Eur. J. Personal..

[12] Lynn R., Hampson S.L. (1975). National differences in extraversion and neuroticism. Br. J. Soc. Clin. Psychol..

[13] Oishi S., Talhelm T., Lee M. (2015). Personality and geography: Introverts prefer mountains. J. Res. Personal..

[14] Berry D.S., Jones G.M., Kuczaj S.A. (2000). Differing states of mind: Regional affiliation, personality judgment, and self-view. Basic Appl. Soc. Psychol..

[15] Shuttleworth I., Stevenson C., Bjarnason Þ., Finell E. (2021). Geography, psychology and the ‘Big Five’personality traits: Who moves, and over what distances, in the United Kingdom?. Popul. Space Place.

[16] Campbell R.D. (1968). Personality as an element of regional geography. Ann. Assoc. Am. Geogr..

[17] Digman J.M. (1990). Personality structure: Emergence of the five-factor model. Annu. Rev. Psychol..

[18] Hofstee W.K., De Raad B., Goldberg L.R. (1992). Integration of the big five and circumplex approaches to trait structure. J. Personal. Soc. Psychol..

[19] Ashton M.C., Lee K., Perugini M., Szarota P., de Vries R.E., Di Blas L., Boies K., De Raad B. (2004). A Six-Factor Structure of Personality-Descriptive Adjectives: Solutions From Psycholexical Studies in Seven Languages. J. Personal. Soc. Psychol..

[20] Ashton M.C., Lee K. (2007). Empirical, theoretical, and practical advantages of the HEXACO model of personality structure. Personal. Soc. Psychol. Rev..

[21] Eysenck H.J. (1992). Four ways five factors are not basic. Personal. Individ. Differ..

[22] Kraczla M. (2017). Osobowość jako czynnik zachowań menedżerskich w świetle teorii Wielkiej Piątki. Zesz. Naukowe. Organ. I Zarządzanie/Politech. Śląska.

[23] Zimbardo P.G., Johnson R.L., McCann V. (2010). Psychologia. Kluczowe Koncepcje.

[24] Lynn R., Martin T. (1995). National differences for thirty-seven nations in extraversion, neuroticism, psychoticism and economic, demographic and other correlates. Personal. Individ. Differ..

[25] Hofstede G. (2001). Culture’s Consequences: Comparing Values, Behaviors, Institutions, and Organizations across Nations.

[26] Riaz M.N., Riaz M.A., Batool N. (2012). Personality Types as Predictors of Decision Making Styles. J. Behav. Sci..

[27] Gopal C.R. (2020). Relationship between personality and decision making styles among college students. Ann. Trop. Med. Public Health.

[28] Markiewicz K., Kaczmarek B.L., Kostka-Szymańska M. (2010). Cechy osobowości a decyzje adolescentów dotyczące planów edukacyjnych i zawodowych. Psychol. Rozw..

[29] Bayram N., Aydemir M. (2017). Decision-making styles and personality traits. Int. J. Recent Adv. Organ. Behav. Decis. Sci..

[30] Dewberry C., Juanchich M., Narendran S. (2013). Decision-making competence in everyday life: The roles of general cognitive styles, decision-making styles and personality. Personal. Individ. Differ..

[31] Gambetti E., Giusberti F. (2019). Personality, decision-making styles and investments. J. Behav. Exp. Econ..

[32] Ju U., Kang J., Wallraven C. (2019). To brake or not to brake? Personality traits predict decision-making in an accident situation. Front. Psychol..

[33] Mendes F., Mendes E., Salleh N., Oivo M. (2021). Insights on the relationship between decision-making style and personality in software engineering. Inf. Softw. Technol..

[34] Weller J., Ceschi A., Hirsch L., Sartori R., Costantini A. (2018). Accounting for individual differences in decision-making competence: Personality and gender differences. Front. Psychol..

[35] Bag S., Omrane A. (2021). The relationship between the personality traits of entrepreneurs and their decision-making process: The role of manufacturing SMEs’ Institutional Environment in India. Forum Sci. Oeconomia.

[36] Soane E., Chmiel N. (2005). Are risk preferences consistent?: The influence of decision domain and personality. Personal. Individ. Differ..

[37] Mendes F.F., Mendes E., Salleh N. (2019). The relationship between personality and decision-making: A Systematic literature review. Inf. Softw. Technol..

[38] Villafaina S., Collado-Mateo D., Cano-Plasencia R., Gusi N., Fuentes J.P. (2019). Electroencephalographic response of chess players in decision-making processes under time pressure. Physiol. Behav..

[39] Amidzic O., Riehle H.J., Elbert T. (2006). Toward a psychophysiology of expertise: Focal magnetic gamma bursts as a signature of memory chunks and the aptitude of chess players. J. Psychophysiol..

[40] Campitelli G., Gobet F. (2004). Adaptive expert decision making: Skilled chess players search more and deeper. ICGA J..

[41] Józefacka-Szram N., Marszałek D. (2018). Szachy w edukacji szkolnej–sposób na poprawę kompetencji i umiejętności przydatnych w szkole i w życiu. Niepełnosprawność.

[42] Franklin G.L., Pereira B.N., Lima N.S., Germiniani F.M.B., Camargo C.H.F., Caramelli P., Teive H.A.G. (2020). Neurology, psychiatry and the chess game: A narrative review. Arq. De Neuro-Psiquiatr..

[43] Blanch A. (2020). Chess and Individual Differences.

[44] Vollstädt-Klein S., Grimm O., Kirsch P., Bilalić M. (2010). Personality of elite male and female chess players and its relation to chess skill. Learn. Individ. Differ..

[45] Grabner R.H., Stern E., Neubauer A.C. (2007). Individual differences in chess expertise: A psychometric investigation. Acta Psychol..

[46] Blanch A., Llaveria A. (2021). Ability and non-ability traits in chess skill. Personal. Individ. Differ..

[47] Gränsmark P. (2012). Masters of our time: Impatience and self-control in high-level chess games. J. Econ. Behav. Organ..

[48] Bilalić M., McLeod P., Gobet F. (2007). Personality profiles of young chess players. Personal. Individ. Differ..

[49] Albarracín D., Wyer R.S. (2000). The cognitive impact of past behavior: Influences on beliefs, attitudes, and future behavioral decisions. J. Personal. Soc. Psychol..

[50] Unterrainer J.M., Kaller C.P., Halsband U., Rahm B. (2006). Planning abilities and chess: A comparison of chess and non-chess players on the Tower of London task. Br. J. Psychol..

[51] Calderwood R., Klein G.A., Crandall B.W. (1988). Time pressure, skill, and move quality in chess. Am. J. Psychol..

[52] Van Harreveld F., Wagenmakers E.J., Van Der Maas H.L. (2007). The effects of time pressure on chess skill: An investigation into fast and slow processes underlying expert performance. Psychol. Res..

[53] Burns B.D. (2004). The effects of speed on skilled chess performance. Psychol. Sci..

[54] Chabris C.F., Hearst E.S. (2003). Visualization, pattern recognition, and forward search: Effects of playing speed and sight of the position on grandmaster chess errors. Cogn. Sci..

[55] Gobet F., Simon H.A. (1996). The roles of recognition processes and look-ahead search in time-constrained expert problem solving: Evidence from grand-master-level chess. Psychol. Sci..

[56] Medvegy Z., Raab M., Tóth K., Csurilla G., Sterbenz T. (2022). When do expert decision makers trust their intuition?. Appl. Cogn. Psychol..

[57] Miric M., Lu J., Teodoridis F. (2020). Decision-Making Skills in an AI World: Lessons from Online Chess. SSRN Electron. J..

[58] Dilmaghani M. (2021). The gender gap in competitive chess across countries: Commanding queens in command economies. J. Comp. Econ..

[59] Dilmaghani M. (2020). Gender differences in performance under time constraint: Evidence from chess tournaments. J. Behav. Exp. Econ..

[60] Stafford T. (2018). Female chess players outperform expectations when playing men. Psychol. Sci..

[61] Dreber A., Gerdes C., Gränsmark P. (2013). Beauty queens and battling knights: Risk taking and attractiveness in chess. J. Econ. Behav. Organ..

[62] Linnemer L., Visser M. (2016). Selection in Tournaments: The Case of Chess Players. J. Econ. Behav. Organ..

[63] Fuentes-García J.P., Patiño M.J.M., Villafaina S., Clemente-Suárez V.J. (2020). The effect of COVID−19 confinement in behavioral, psychological, and training patterns of chess players. Front. Psychol..

[64] Vishkin A. (2022). Queen’s gambit declined: The gender-equality paradox in chess participation across 160 countries. Psychol. Sci..

[65] Gruchman G. Cel Zaklęty w Miernikach. ComputerWorld 2002. https://www.computerworld.pl/porada/Cel-zaklety-w-miernikach,293796.html.

[66] Elo A.E. (1978). The Rating of Chessplayers, Past and Present.

[67] Van Der Maas H.L., Wagenmakers E.J. (2005). A psychometric analysis of chess expertise. Am. J. Psychol..

[68] Robinson A.A. (2021). Teaching AI to Play Chess Like People. https://umm-csci.github.io/senior-seminar/seminars/spring2021/robinson.pdf.

[69] Roy R. Monte Carlo Tree Search. 2019 (MCTS). https://www.geeksforgeeks.org/ml-monte-carlo-treesearch-mcts/.

[70] Silver D., Hubert T., Schrittwieser J., Antonoglou I., Lai M., Guez A., Lanctot M., Sifre L., Kumaran D., Graepel T. (2018). A general reinforcement learning algorithm that masters chess, shogi, and go through self-play. Science.

[71] Leśniczek A. (2011). Supporting learning processes of pre-school children. The use of educational kinesiology in an effective learning–be the master of your mind. Pr. Nauk. Akad. Jana Długosza Częstochowie. Studia Neofilologiczne.

[72] Noir M. (2002). Le Développement des Habiletés Cognitives de L’enfant par la Pratique du jeu D’échecs: Essai de Modélisation d’une Didactique du Transfert. Ph.D. Thesis.

[73] Trinchero R. Chess as a Cognitive Training Ground. Six Years of Trials in Primary Schools. https://www.europechesspromotion.org/upload/pagine/doc/Chess%20researches%202005−2011%20Trinchero-Romano%20-%20Martini.pdf.

