# Supplementary material for: The Theoretical Model of Decision-Making Behaviour Geospatial Analysis Using Data Obtained from the Games of Chess
Source: Int J Environ Res Public Health. 2022 Sep 28;19(19):12353. doi: 10.3390/ijerph191912353 (PMC9566314; doi:10.3390/ijerph191912353)
Supplement: Supplementary file 1 [file ijerph-19-12353-s001.zip › ijerph-1894318-supplementary.pdf]

Supplementary

Cofiguration of possible combination of information - examples

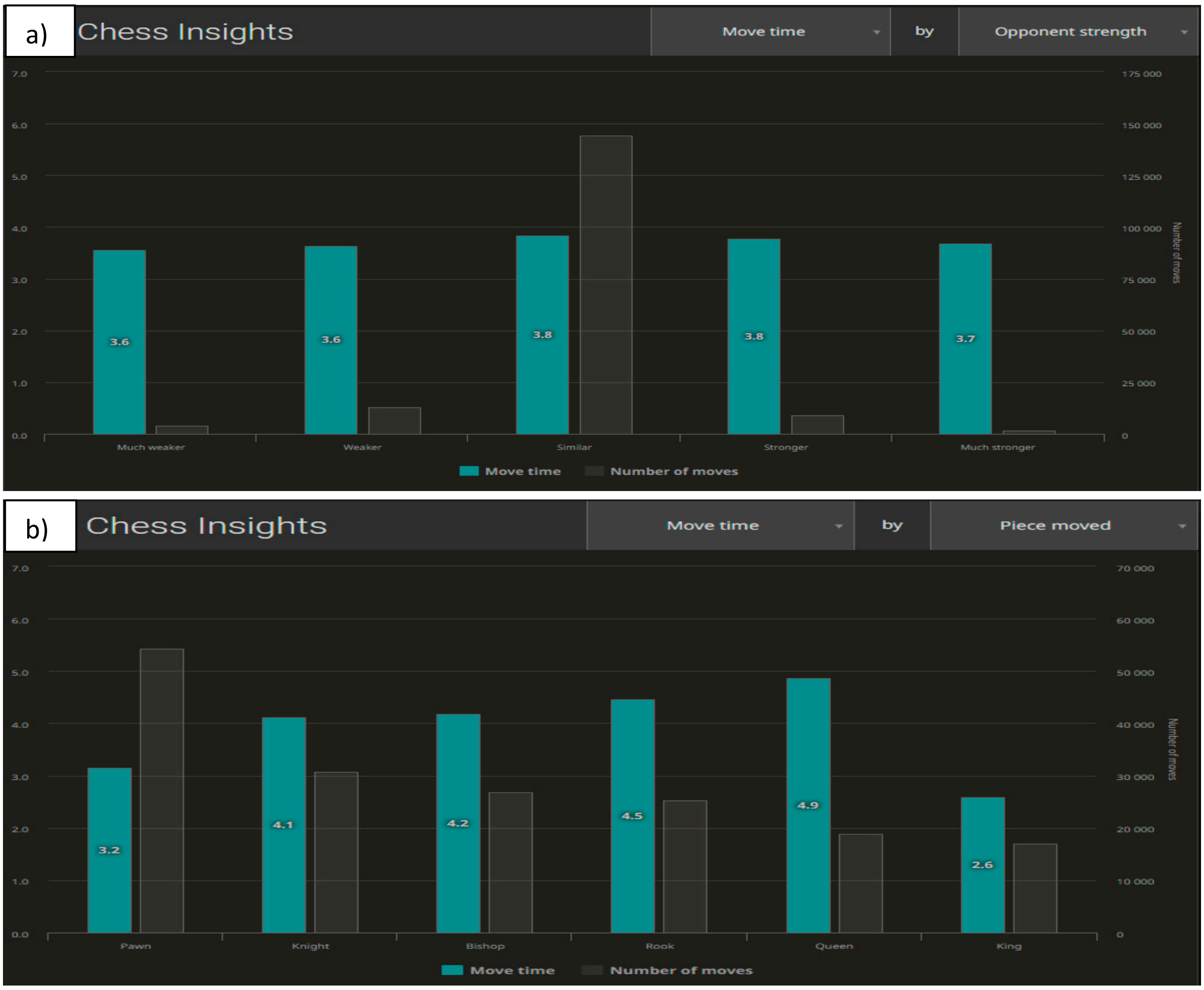

Figure S1. Correlation of the time spent per move taking into account the strength of the opponents - a) and the average time spent making a move with an individual piece - b). Source: lichess.org

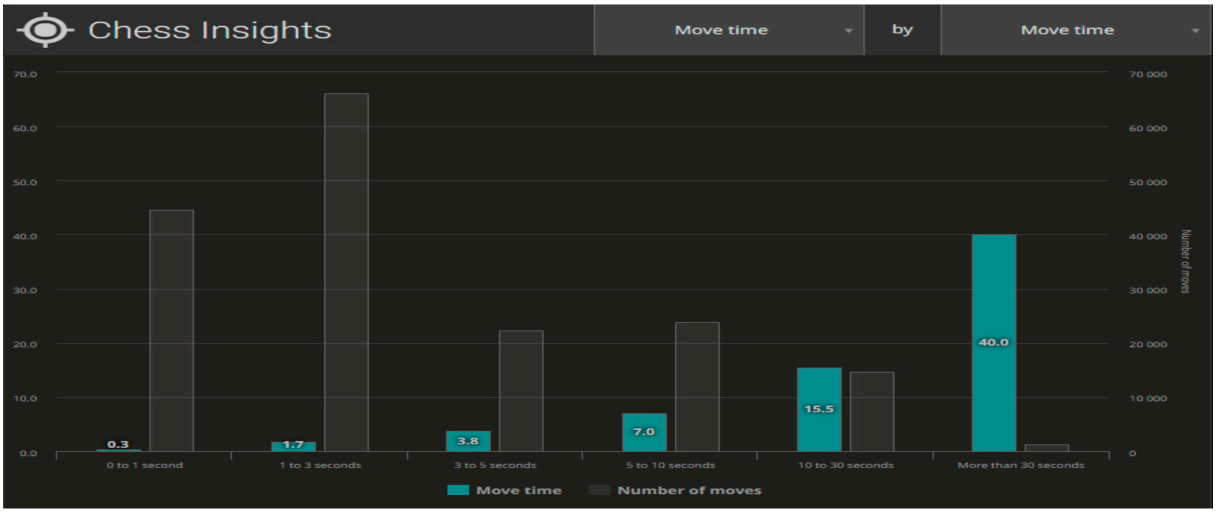

Figure S2. Statistics concerning the number of moves in each time slot. Source: lichess.org

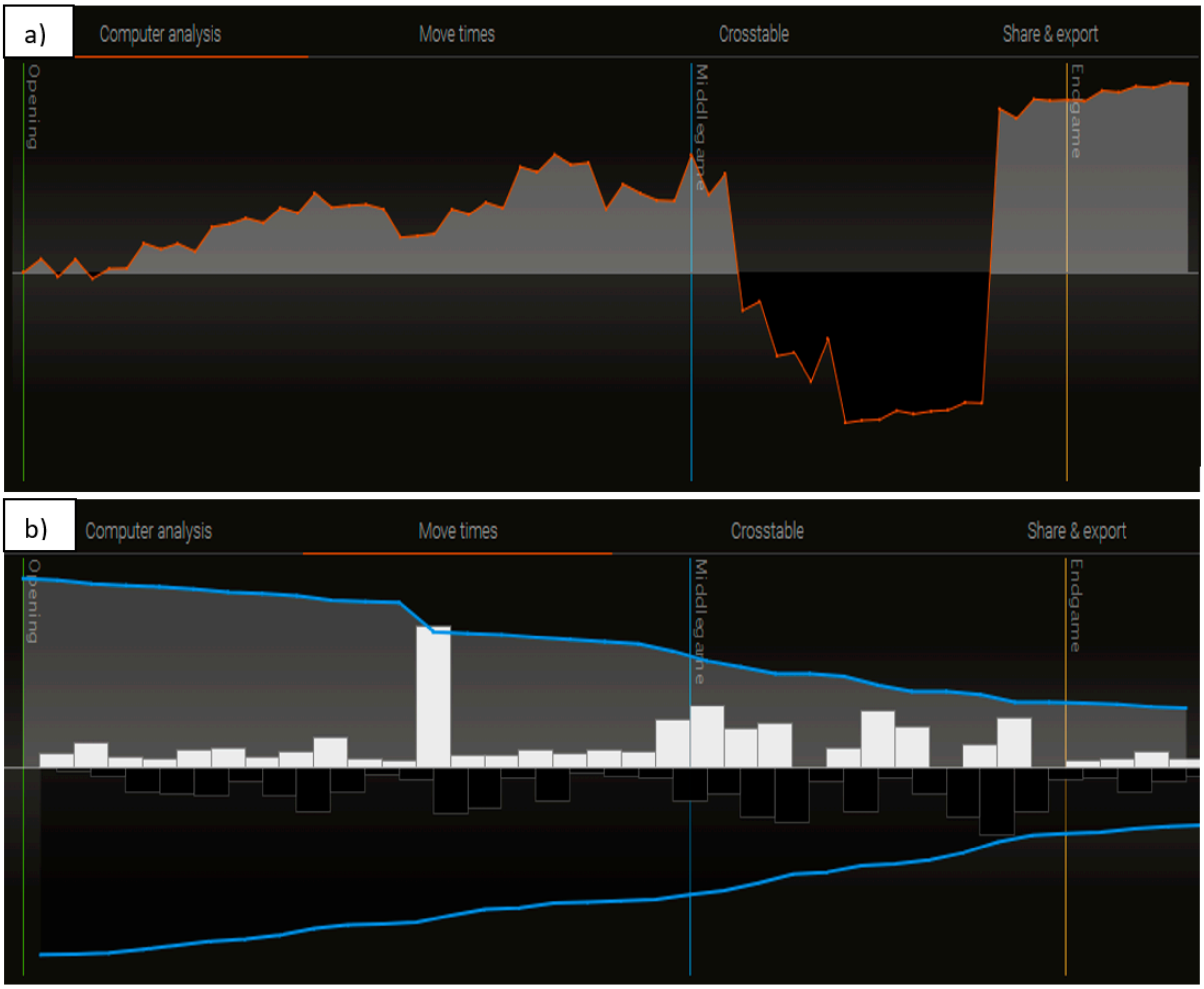

Figure S3. The analysis of a single game using the chess engine - a) and the statistical analysis that takes into account the time spent per move - b). Source: lichess.org

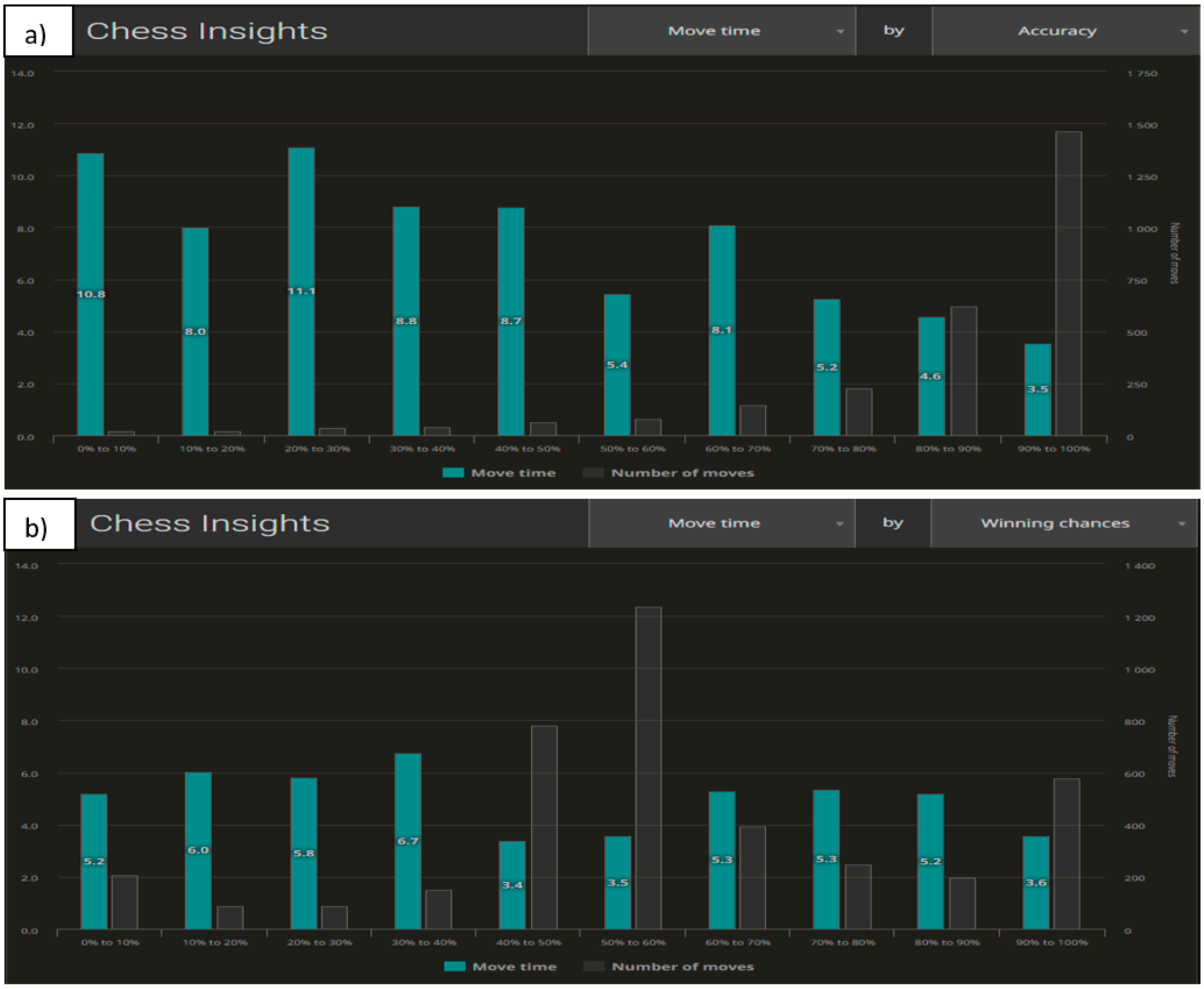

Figure S4. Evaluation of the quality of the decisions made, with regard to the average time per move - a), and evaluation of the chances of winning in relation to the time spent per move - b). Source: lichess.org

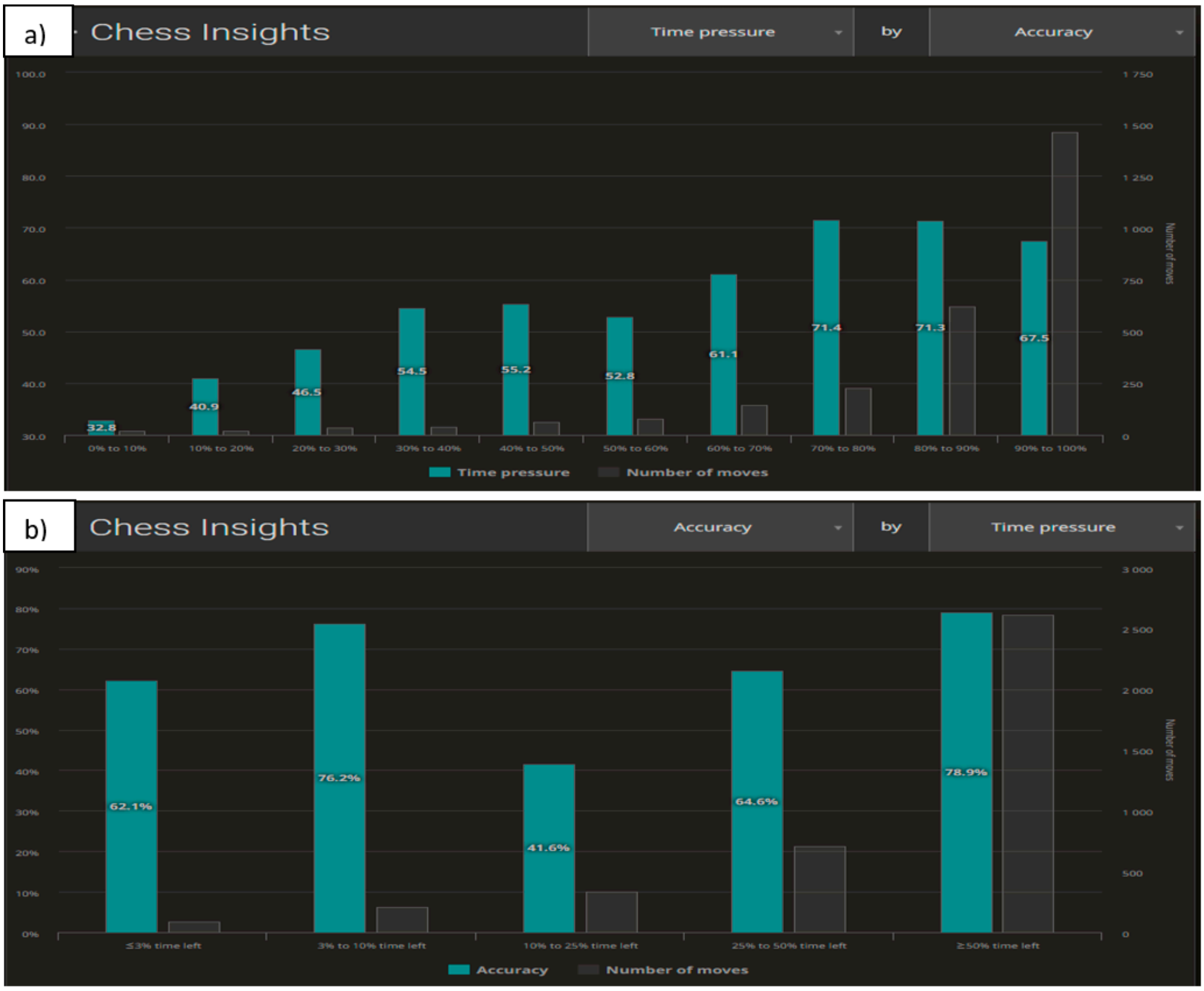

Figure S5. Correlation of time pressure and accuracy of decision making (against chess engine evaluation) - a) and overview showing accuracy with time pressure taken into account - b). Source: lichess.org

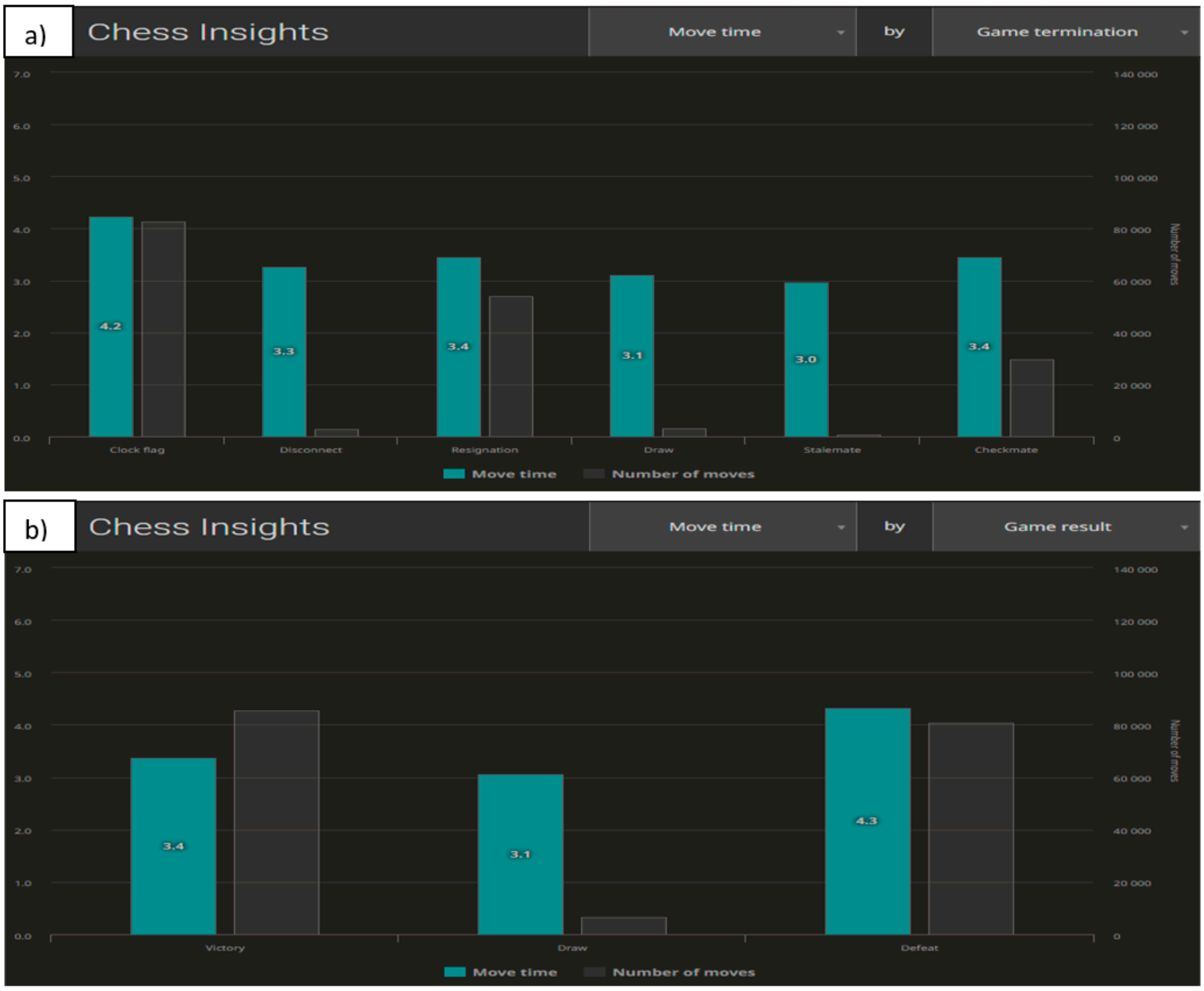

Figure S6. Game termination type statistics - a) and game result statistics - b). Source: lichess.org

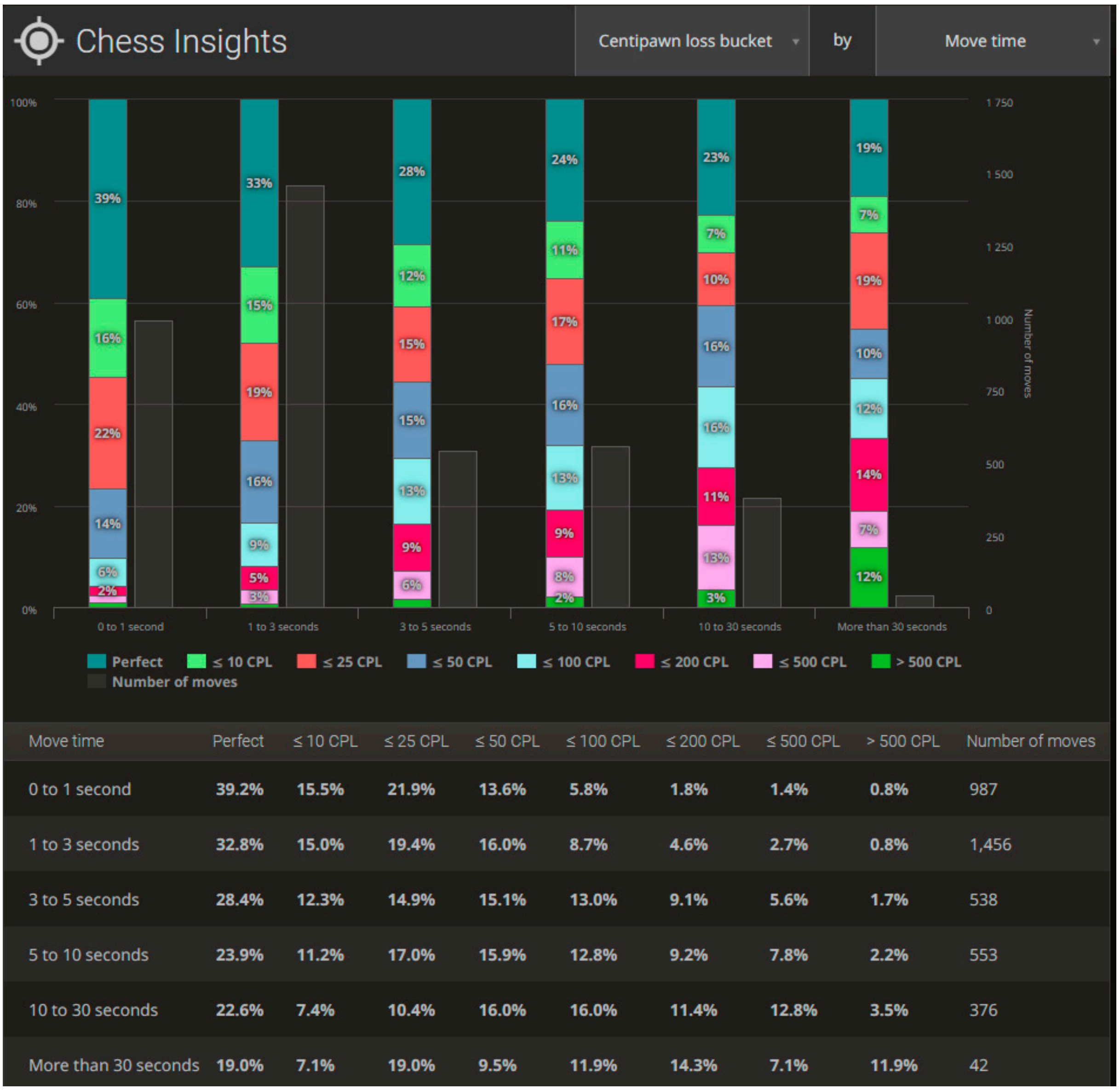

Move time	Perfect	≤ 10 CPL	≤ 25 CPL	≤ 50 CPL	≤ 100 CPL	≤ 200 CPL	≤ 500 CPL	> 500 CPL	Number of moves
0 to 1 second	39.2%	15.5%	21.9%	13.6%	5.8%	1.8%	1.4%	0.8%	987
1 to 3 seconds	32.8%	15.0%	19.4%	16.0%	8.7%	4.6%	2.7%	0.8%	1,456
3 to 5 seconds	28.4%	12.3%	14.9%	15.1%	13.0%	9.1%	5.6%	1.7%	538
5 to 10 seconds	23.9%	11.2%	17.0%	15.9%	12.8%	9.2%	7.8%	2.2%	553
10 to 30 seconds	22.6%	7.4%	10.4%	16.0%	16.0%	11.4%	12.8%	3.5%	376
More than 30 seconds	19.0%	7.1%	19.0%	9.5%	11.9%	14.3%	7.1%	11.9%	42

Figure S7. Correlations between Centipawn loss bucket and Move time. Source: lichess.org

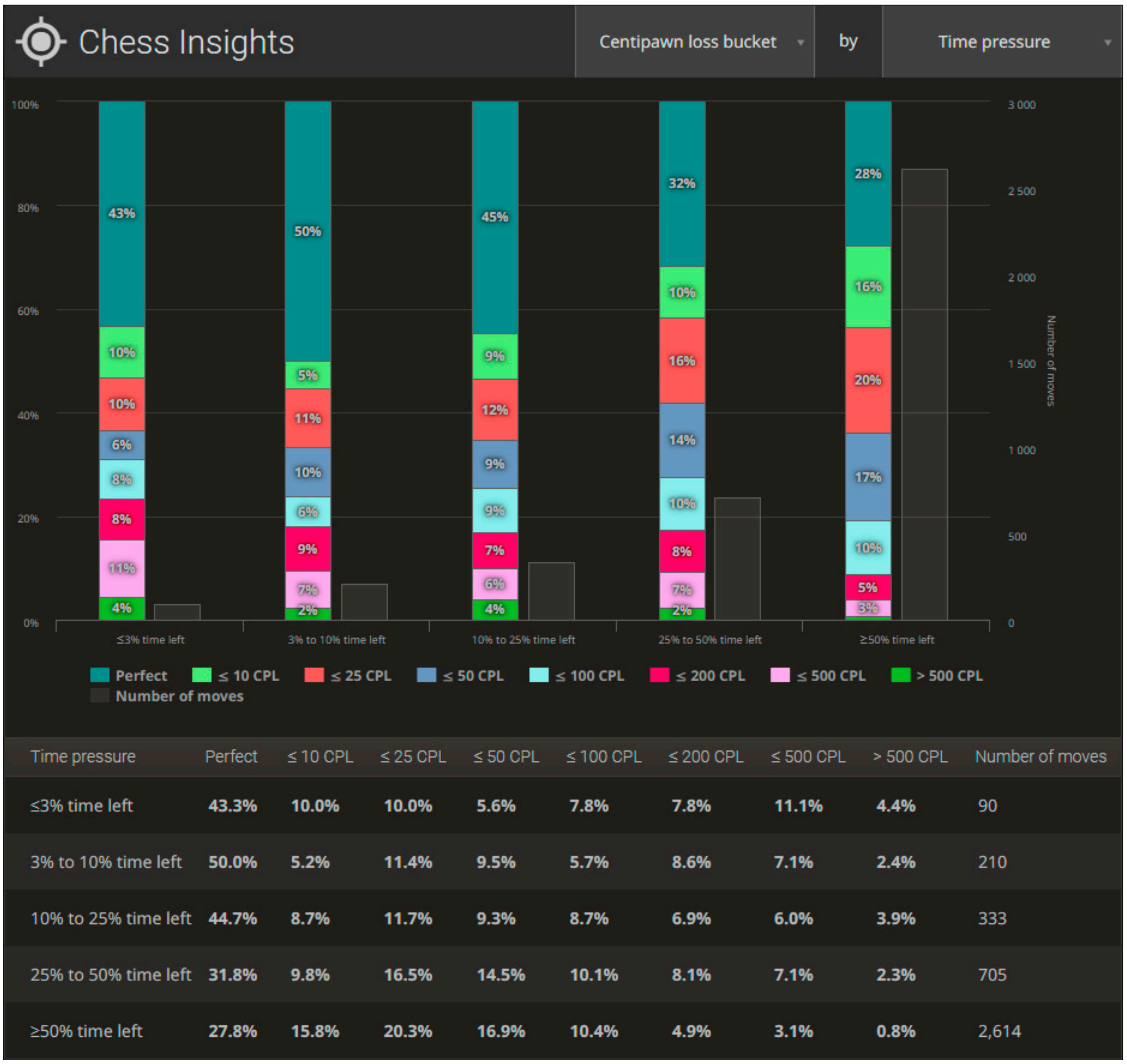

Time pressure	Perfect	≤ 10 CPL	≤ 25 CPL	≤ 50 CPL	≤ 100 CPL	≤ 200 CPL	≤ 500 CPL	> 500 CPL	Number of moves
≤3% time left	**43.3%**	**10.0%**	**10.0%**	**5.6%**	**7.8%**	**7.8%**	**11.1%**	**4.4%**	90
3% to 10% time left	**50.0%**	**5.2%**	**11.4%**	**9.5%**	**5.7%**	**8.6%**	**7.1%**	**2.4%**	210
10% to 25% time left	**44.7%**	**8.7%**	**11.7%**	**9.3%**	**8.7%**	**6.9%**	**6.0%**	**3.9%**	333
25% to 50% time left	**31.8%**	**9.8%**	**16.5%**	**14.5%**	**10.1%**	**8.1%**	**7.1%**	**2.3%**	705
≥50% time left	**27.8%**	**15.8%**	**20.3%**	**16.9%**	**10.4%**	**4.9%**	**3.1%**	**0.8%**	2,614

Figure S8. Correlations between Centipawn loss bucket and time pressure. Source: lichess.org

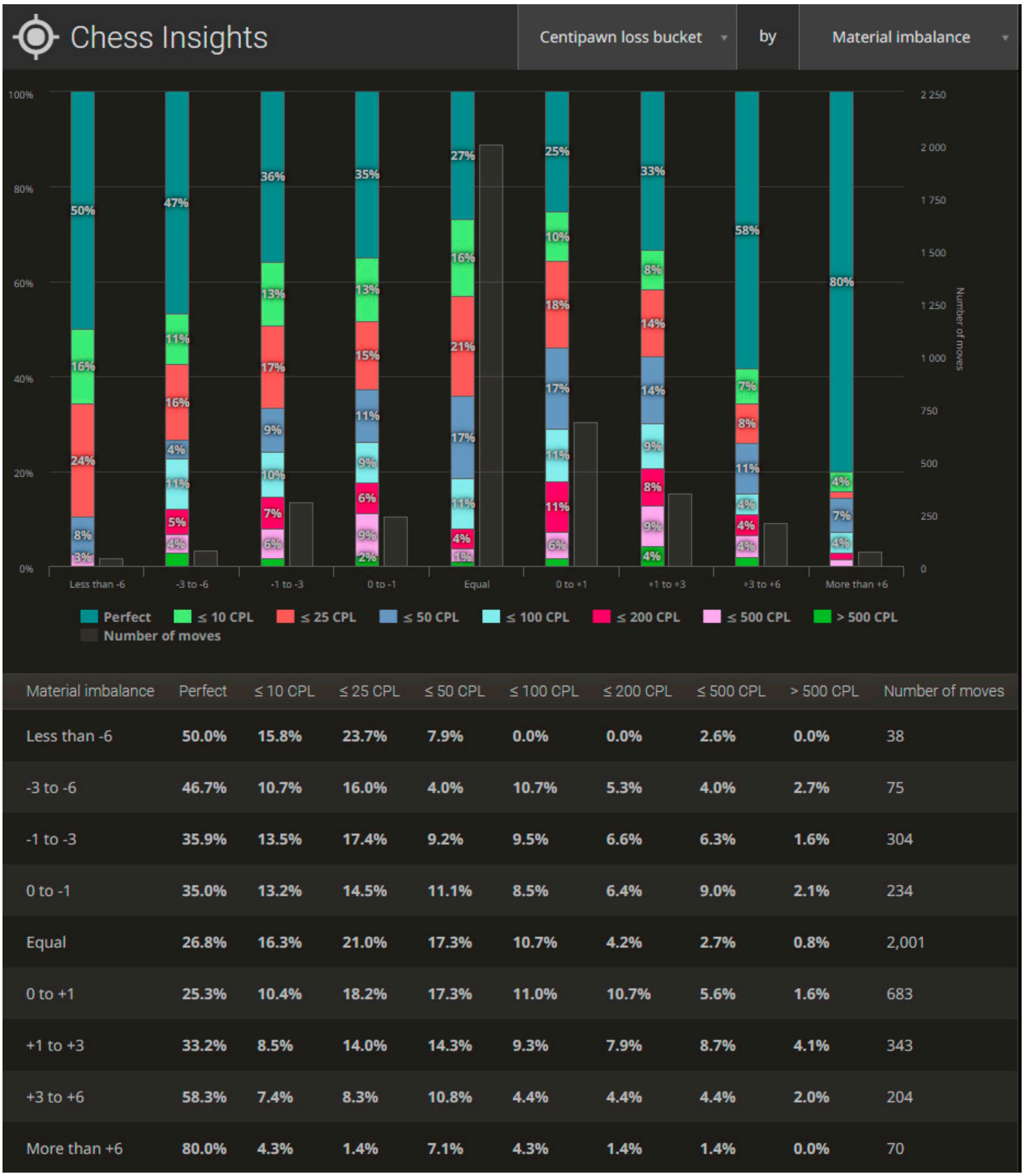

Material imbalance	Perfect	≤ 10 CPL	≤ 25 CPL	≤ 50 CPL	≤ 100 CPL	≤ 200 CPL	≤ 500 CPL	> 500 CPL	Number of moves
Less than -6	50.0%	15.8%	23.7%	7.9%	0.0%	0.0%	2.6%	0.0%	38
-3 to -6	46.7%	10.7%	16.0%	4.0%	10.7%	5.3%	4.0%	2.7%	75
-1 to -3	35.9%	13.5%	17.4%	9.2%	9.5%	6.6%	6.3%	1.6%	304
0 to -1	35.0%	13.2%	14.5%	11.1%	8.5%	6.4%	9.0%	2.1%	234
Equal	26.8%	16.3%	21.0%	17.3%	10.7%	4.2%	2.7%	0.8%	2,001
0 to +1	25.3%	10.4%	18.2%	17.3%	11.0%	10.7%	5.6%	1.6%	683
+1 to +3	33.2%	8.5%	14.0%	14.3%	9.3%	7.9%	8.7%	4.1%	343
+3 to +6	58.3%	7.4%	8.3%	10.8%	4.4%	4.4%	4.4%	2.0%	204
More than +6	80.0%	4.3%	1.4%	7.1%	4.3%	1.4%	1.4%	0.0%	70

Figure S9. Correlations between Centipawn loss bucket and Material imbalance. Source: lichess.org

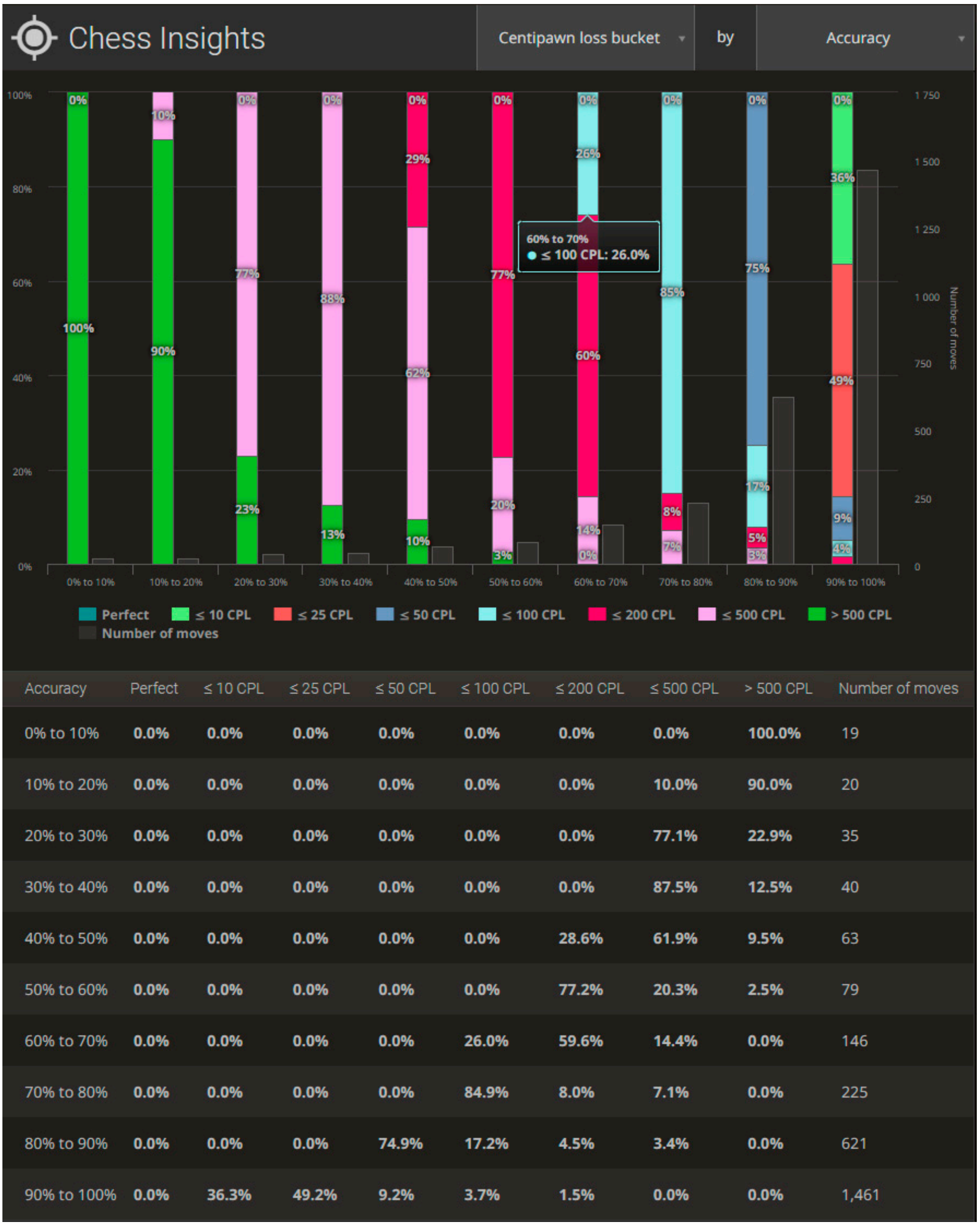

Accuracy	Perfect	≤ 10 CPL	≤ 25 CPL	≤ 50 CPL	≤ 100 CPL	≤ 200 CPL	≤ 500 CPL	> 500 CPL	Number of moves
0% to 10%	0.0%	0.0%	0.0%	0.0%	0.0%	0.0%	0.0%	100.0%	19
10% to 20%	0.0%	0.0%	0.0%	0.0%	0.0%	0.0%	10.0%	90.0%	20
20% to 30%	0.0%	0.0%	0.0%	0.0%	0.0%	0.0%	77.1%	22.9%	35
30% to 40%	0.0%	0.0%	0.0%	0.0%	0.0%	0.0%	87.5%	12.5%	40
40% to 50%	0.0%	0.0%	0.0%	0.0%	0.0%	28.6%	61.9%	9.5%	63
50% to 60%	0.0%	0.0%	0.0%	0.0%	0.0%	77.2%	20.3%	2.5%	79
60% to 70%	0.0%	0.0%	0.0%	0.0%	26.0%	59.6%	14.4%	0.0%	146
70% to 80%	0.0%	0.0%	0.0%	0.0%	84.9%	8.0%	7.1%	0.0%	225
80% to 90%	0.0%	0.0%	0.0%	74.9%	17.2%	4.5%	3.4%	0.0%	621
90% to 100%	0.0%	36.3%	49.2%	9.2%	3.7%	1.5%	0.0%	0.0%	1,461

Figure S10. Correlations between Centipawn loss bucket and Accuracy. Source: lichess.org

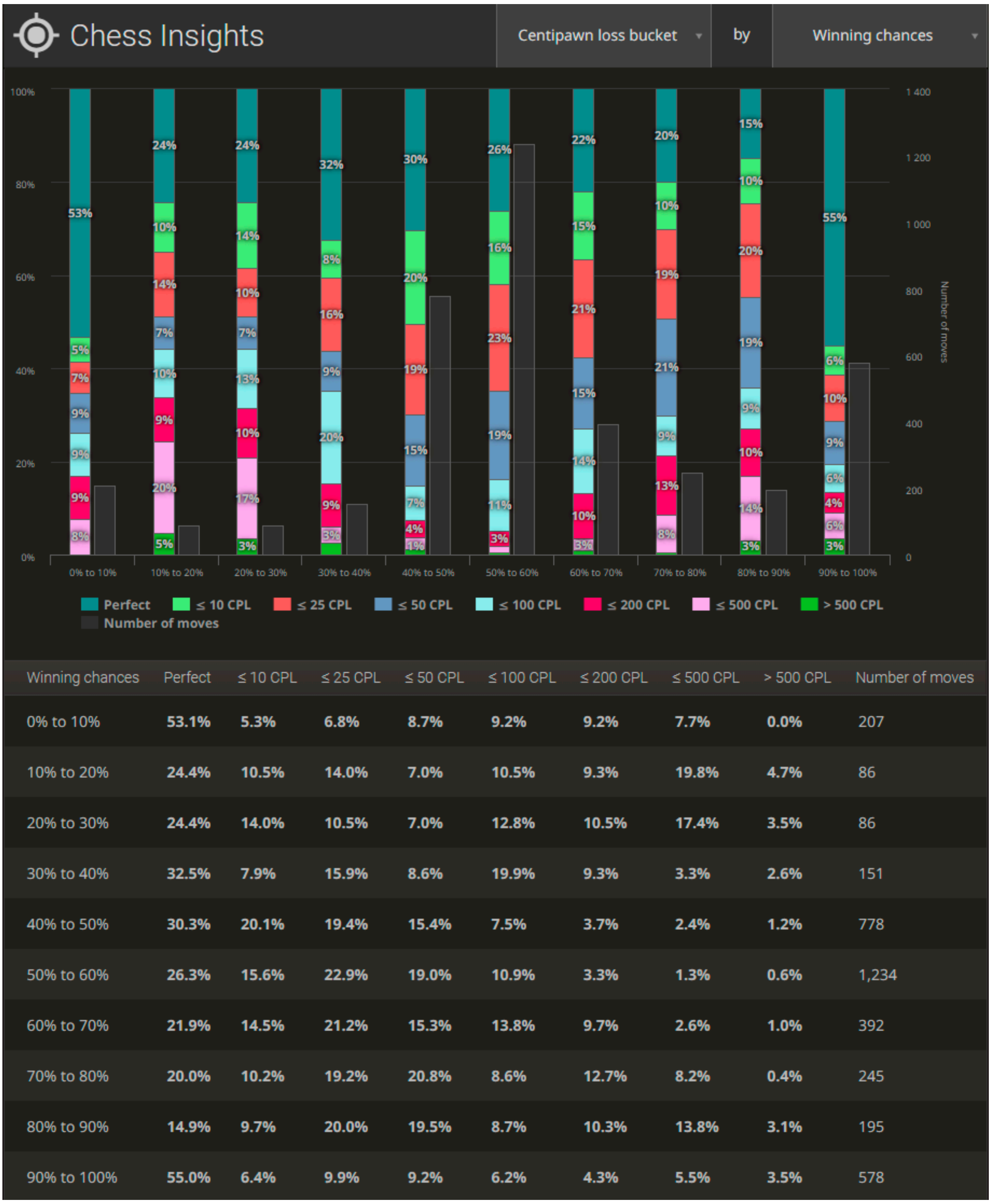

Winning chances	Perfect	≤ 10 CPL	≤ 25 CPL	≤ 50 CPL	≤ 100 CPL	≤ 200 CPL	≤ 500 CPL	> 500 CPL	Number of moves
0% to 10%	53.1%	5.3%	6.8%	8.7%	9.2%	9.2%	7.7%	0.0%	207
10% to 20%	24.4%	10.5%	14.0%	7.0%	10.5%	9.3%	19.8%	4.7%	86
20% to 30%	24.4%	14.0%	10.5%	7.0%	12.8%	10.5%	17.4%	3.5%	86
30% to 40%	32.5%	7.9%	15.9%	8.6%	19.9%	9.3%	3.3%	2.6%	151
40% to 50%	30.3%	20.1%	19.4%	15.4%	7.5%	3.7%	2.4%	1.2%	778
50% to 60%	26.3%	15.6%	22.9%	19.0%	10.9%	3.3%	1.3%	0.6%	1,234
60% to 70%	21.9%	14.5%	21.2%	15.3%	13.8%	9.7%	2.6%	1.0%	392
70% to 80%	20.0%	10.2%	19.2%	20.8%	8.6%	12.7%	8.2%	0.4%	245
80% to 90%	14.9%	9.7%	20.0%	19.5%	8.7%	10.3%	13.8%	3.1%	195
90% to 100%	55.0%	6.4%	9.9%	9.2%	6.2%	4.3%	5.5%	3.5%	578

Figure S11. Correlations between Centipawn loss bucket and Winning chances. Source: lichess.org

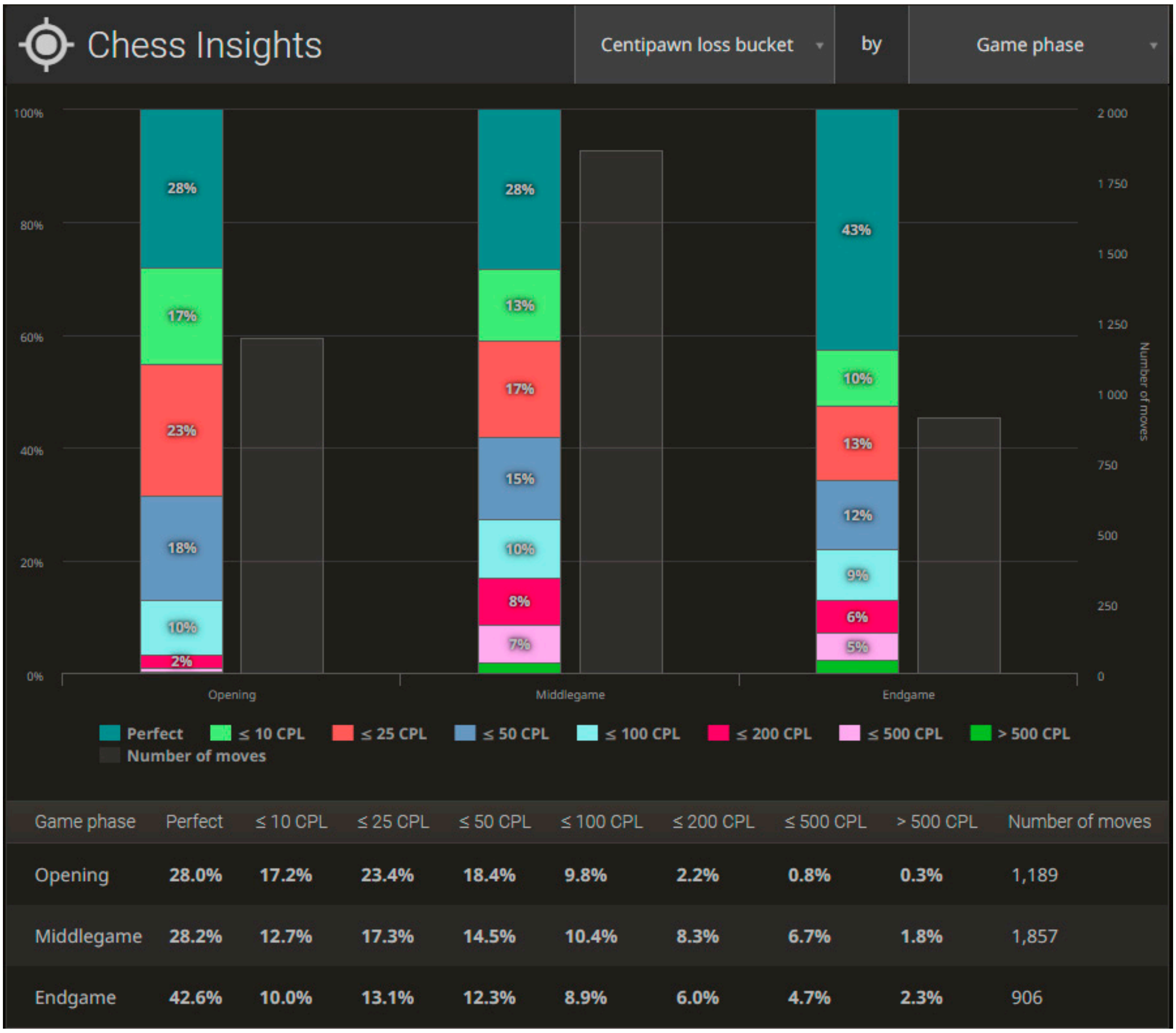

Game phase	Perfect	≤ 10 CPL	≤ 25 CPL	≤ 50 CPL	≤ 100 CPL	≤ 200 CPL	≤ 500 CPL	> 500 CPL	Number of moves
Opening	28.0%	17.2%	23.4%	18.4%	9.8%	2.2%	0.8%	0.3%	1,189
Middlegame	28.2%	12.7%	17.3%	14.5%	10.4%	8.3%	6.7%	1.8%	1,857
Endgame	42.6%	10.0%	13.1%	12.3%	8.9%	6.0%	4.7%	2.3%	906

Figure S12. Correlations between Centipawn loss bucket and Game phase. Source: lichess.org
